# Visual Parameter Space Exploration in Time and Space

**DOI:** 10.1111/cgf.14785

**Published:** 2023-04-03

**Authors:** Nikolaus Piccolotto, Markus Bögl, Silvia Miksch

**Affiliations:** ^1^ TU Wien Institute of Visual Computing and Human‐Centered Technology Wien Austria

**Keywords:** parameter space analysis, visual analytics, visualization

## Abstract

Computational models, such as simulations, are central to a wide range of fields in science and industry. Those models take input parameters and produce some output. To fully exploit their utility, relations between parameters and outputs must be understood. These include, for example, which parameter setting produces the best result (optimization) or which ranges of parameter settings produce a wide variety of results (sensitivity). Such tasks are often difficult to achieve for various reasons, for example, the size of the parameter space, and supported with visual analytics. In this paper, we survey visual parameter space exploration (VPSE) systems involving spatial and temporal data. We focus on interactive visualizations and user interfaces. Through thematic analysis of the surveyed papers, we identify common workflow steps and approaches to support them. We also identify topics for future work that will help enable VPSE on a greater variety of computational models.

## Graphical abstract

In our survey of visual parameter space exploration with spatial/temporal data, we identify five themes forming the components of a common workflow.

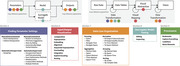

## 1 Introduction

Computational models, like simulations, data mining, or generative/procedural models, are indispensable to modern science and industry. However, to fully harness their utility, analysts must understand the model's parameters and find adequate parameter settings, which poses complex challenges. Computational models often work on spatial and temporal data [STBB14, CSS*19, WHLS19, MT20]. In meteorology, simulation models of the atmosphere are used to predict precipitation and extreme weather conditions [WLSL17]. Architects employ finite element analysis to ensure the load‐bearing walls are durable [SMS*17]. Generative models support 3D artists to design geometries of different scales, from coffee mugs [BHGK14] to whole cities [VGA*12]. Data mining models, such as image segmentation algorithms, have their use in manufacturing to assess material porosity [WAG*16], as well as in medicine, where they separate tissue types [PZR15]. The canon in visualization literature is that spatial and temporal data have unique properties (e.g., [Hai09; Mun14, p. 28]) and, therefore, should be visualized as such and may not be treated as some other numeric variables. Due to their unique character, we focus this survey on papers where the model's parameters or output reference or exist in time/space.

For our purposes, we consider all such models as input/output models: Some input fed into the model generates some output. Inputs can be control/model parameters [SWN03], like thresholds/weights, as well as other data the model works on, for example, an image in the case of image segmentation. We discuss this in more detail in Section 2. Parameter space analysis tasks [SHB*14] often involve analysis of relations between the model's parameters and outputs. For example, if small changes in a parameter lead to significant changes in output (*sensitivity* analysis), which parameters lead to optimal output based on some objectives (*optimization*), or which parameters produce the most reliable output (*uncertainty*). We collect all tasks under the term “visual parameter space exploration” (VPSE).

VPSE is a prime example of visual analytics [TC05, KAF*08], where visual and automatic methods are combined, thus leveraging the human's and computer's individual strengths. VPSE is also a relatively mature sub‐field of visualization and visual analytics. Seminal works were published in the 1990s, like Design Galleries [MBA*97] or spreadsheet interfaces [CBRK97, JKM00]. VPSE has been applied to a broad range of domains, models, and data types, for example, image segmentation [TSM*11], biology simulations [LRHS14], or lighting design [WSL*20], where it proved incredibly useful. Despite the success, the visualization community lacks a systematic review of how user interfaces for VPSE systems work, that is, visualizations, interactions, and available functionality. We believe past systems employed common design elements worth surfacing and classifying. Looking back at successful approaches also often leads to new research directions, which helps us as a community move forward. We intend to fill these gaps with this survey. Our target audiences are visualization designers and researchers working with parameter spaces of computational models. Eliciting and presenting common aspects of VPSE systems is helpful for the former group to evoke a more structured thought process about the problem. It will also allow them to find and compare solutions to visual design problems or choices of automatic techniques in similar contexts. An overview of VPSE systems is advantageous for visualization researchers, who may identify ideas for novel applications or designs more quickly.

The main contributions of our survey are that we

- provide a systematic literature review of VPSE involving spatial and temporal data and focus on the user interface;
- develop common themes in the collected papers by thematic analysis;
- propose a categorization scheme for VPSE works based on the developed themes; and
- outline areas for future research based on the proposed categorization scheme and surveyed papers, such as supporting more parameter space tasks for spatial/temporal parameters, or advanced interactions with parameter spaces.

**Figure 1:**
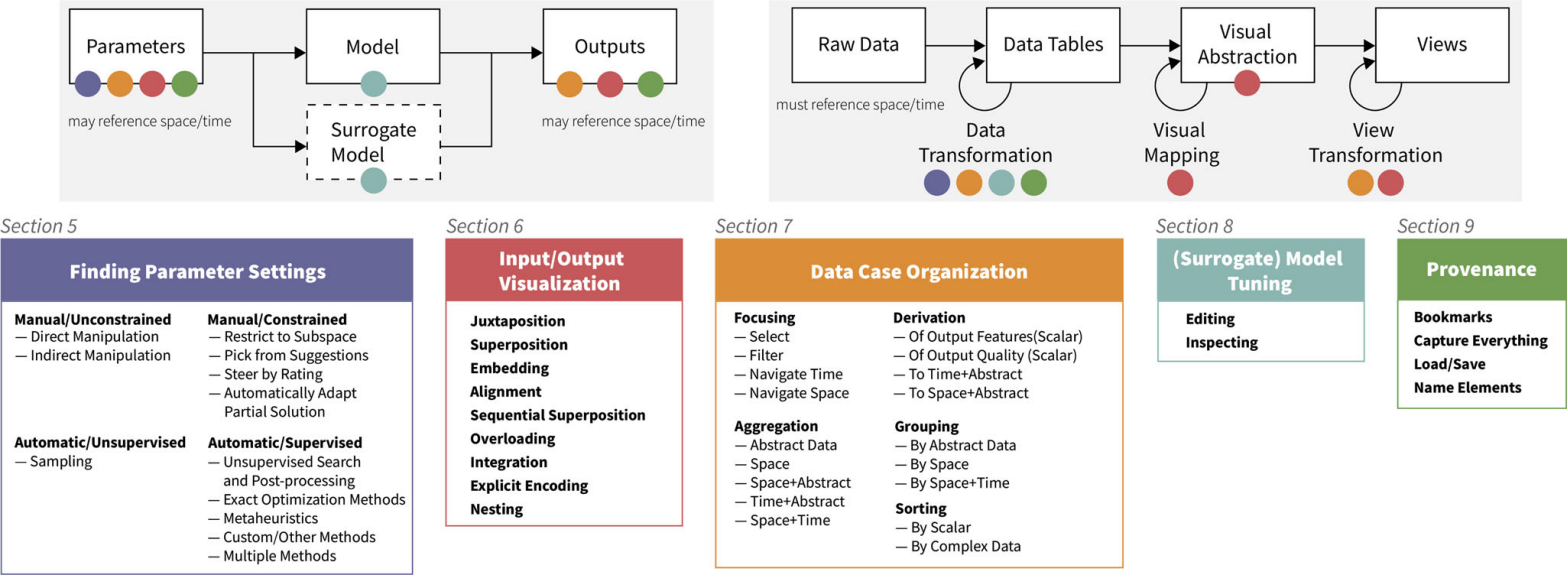
The themes identified as part of our survey describe common actions in a workflow for visual parameter space exploration (VPSE). The relation of our themes to a simplified data flow model in VPSE based on Sedlmair et al. [SHB*14] (left) and the InfoVis pipeline by Card et al. [CMS99] (right) is shown on top. We focus on models where either parameters or outputs reference space and/or time.

The identified themes (Figure 1) describe parts of a VPSE workflow, which we illustrate with an example. Consider a time series segmentation model [BBB*18, EST20]. The model inputs are a multivariate time series, for example, motion sensor data, and some scalar parameters concerning the segmentation process. The model produces a labelled time series, for example, activities. Analysts may look for a reasonable labelling, that is, one that is not overly *sensitive* to particular parameter settings. As a first step, analysts must identify interesting parameter settings to investigate (**Finding Parameter Settings**, Section 5). In this case, the VPSE system computes segmentations for a uniformly random sampling of the parameter space. The obtained parameter/output pairs are then visualized to support the intended analysis (**Input/Output Visualization**, Section 6). For example, parameters and outputs may be shown in a tabular visualization (Figure 14a) using grayscale colour for parameter values and colour hue for labels. Others may depict derived data, like how much changes in a parameter correlate with changes in a label's occurrence (Figure 20b). The analyst then interacts with the visualizations according to current information needs (**Data Case Organization**, Section 7), for example, by zooming into a temporal interval of interest, sorting the table by a column, or defining new derived attributes. In doing so, the analyst formulates hypotheses from gained insights [SSS*14], for example, what a reasonable parameter subspace would be, and acts upon them to verify. This verification may entail changing how the model itself behaves (**(Surrogate) Model Tuning**, Section 8) or repeating the analysis on a smaller parameter subspace. The analyst keeps track of sensible candidates via bookmarking or saving the parameter settings to a file (**Provenance**, Section 9).

The remainder of this paper is structured as follows. In Section 2 we clarify the scope of the survey and the terminology we use. We present related work in Section 3 and describe our method in Section 4. From that point on, we discuss the themes involved in the VPSE workflow in the order they appear in the example above (Sections 5, [Link], [Link], Surrogate Models, Connection to Navigation Strategies, 5.1, [Link], Indirect Manipulation, Direct Manipulation, 5.2, [Link], Restrict to Subspace, Pick from Suggestions, Steer by Rating, Automatically Adapt Partial Solution, 5.3, 5.4, [Link], Unsupervised Search With Post‐Processing, Exact Optimization Methods, Metaheuristics, Custom/Other Methods, Multiple Methods, 6, 6.1, 6.2, 6.3, 6.4, 6.5, 6.6, 6.7, 6.8, 6.9, 7, 7.1, [Link], Select, Filter, Navigate Time, Navigate Space, 7.2, [Link], Scalars Quantifying Output Features, Scalars Quantifying Output Quality, To Time+Abstract Data, To Space+Abstract Data, 7.3, [Link], Abstract Data, Space, Space+Abstract, Time+Abstract, Space+Time, 7.4, [Link], By Scalar, By Complex Attribute, 7.5, [Link], By Abstract Data, By Space, By Space+Time, 8, 8.1, 8.2, 9). A table that shows the distribution of sub‐themes among surveyed papers accompanies every section. We provide illustrations and example figures where applicable. After describing VPSE workflow themes, we discuss relations to other taxonomies (Section 10), present open challenges to the field (Section 11), and close the paper with the conclusion (Section 12).

## 2 Terminology and Scope

**Figure 2:**
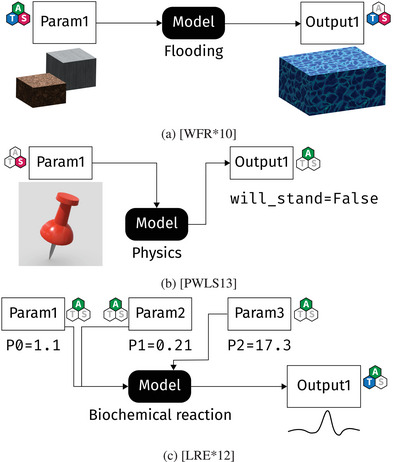
Several examples for models in our survey. (a) Flood simulation: The model takes a 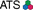 parameter (barriers) and produces a 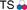 output (water volume). (b) Physics: The model takes a 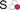 parameter (3D model) and produces an 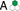 output (whether or not the shape is balanced). (c) Biochemistry: The model takes several 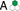 parameters and produces a 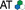 output (number of species over time).

A *model* transforms some input to some output. It can be an existing algorithm, a faster but less accurate “surrogate” to some existing algorithm (usually the case in connection with simulations), or a set of building blocks that perform a specific task, like a processing pipeline. We distinguish between three types of *data cases*: Static inputs (often called *input data*), dynamic inputs (*parameters*), and *output* of a model. The difference between static and dynamic inputs is that the latter take on varying settings to complete a parameter space analysis task [SHB*14], while the other remains static throughout the analysis. We further distinguish between three data characteristics: Spatial (S), temporal (T), and abstract (A) data. Spatial data refers to spatial primitives, like points or volumes. Temporal data refers to temporal primitives, like instants or intervals, and abstract data to tensors. Spatially and temporally varying data arises by combining the three characteristics, for example, a multivariate time series has both temporal and abstract features as an associated vector of variables exists for each time instant. Abstract, spatial, and temporal characteristics amount to seven possible combinations, which we denote by a glyph of three hexagons, for example, 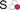 for spatial data (also compare Figure 2).

The scope of this survey is parameter space analysis tasks for models where *either or both* parameter and output reference time or space. Figure 2 shows a few examples. In flood simulations (Figure 2a) the task of the analyst is to prevent damage to objects of interest, for example, subway entries. The output of the model is the water level, that is, a temporally varying 3D volume, thus it has space and time characteristics (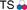). The tools to prevent rising water are barriers, for example, sand bags, which have all characteristics (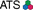): The analyst picks a type of barrier (abstract), places the barrier somewhere (space) and decides when its construction must be completed (time). Figure 2b shows a physics simulation model, where the goal is to design a sculpture that will be in perfect balance [PWLS13]. In other words, it does not fall over. The shape is a 3D volume and thus a spatial parameter (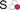), while the output is a Boolean (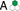) that determines the balance status (whether or not it falls over). Finally, in Figure 2c, a biochemical reaction simulation is considered [LRE*12]. Three numeric parameters (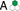) are fed into the simulation, which outputs the number of a given species over time (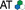). In such a scenario, only the output has a temporal dimension. Hence, our survey includes abstract data on either side of the model as long as the other side has temporal or spatial data.

## 3 Related Work

We will contextualize our survey within the state of the art and existing literature in this section. Regarding surveys in information visualization (InfoVis) in general, McNabb and Laramee provided a survey of surveys [ML19] while Rees and Laramee published a survey of information visualization books [RL19]. In these surveys, more papers about specific interaction idioms or visualizations can be found, that are commonly used in InfoVis in general and VPSE specifically. Such include parallel coordinates [JF16], scatterplots [SG18], summary visualizations [SGS18], uncertainty visualization [BHJ*14], or visual comparison [GAW*11, Gle18]. Surveys about interaction idioms, such as lenses [TGK*17] or focus/context techniques [CKB09], exist too.

Sedlmair et al. [SHB*14] surveyed the literature about visual parameter space analysis, but took a more system‐centric view and do not consider the user interface, like we do. They identified user tasks, like partitioning or sensitivity analysis, as well as navigation strategies in parameter spaces, like informed trial and error, local‐to‐global, global‐to‐local and steering. We discuss the relations to our work in Section 5. Chen et al. [CSS*19] surveyed “Multi‐Space Techniques”, but limited themselves to spatio‐temporal *simulations*, while we take a broader view and also include non‐simulation models. VPSE often goes hand in hand with ensemble visualization (and the other way around), for which Kehrer and Hauser [KH13] as well as Wang et al. [WHLS19] provided surveys.

Our survey focuses on VPSE where parameters or output reference space or time. Many examples exist for VPSE without these, such as HyperMoVal [PBK10], ParaGlide [BSM*13], LineUp [GLG*13], WeightLifter [PST*17] or SenVis [YBP21]. While VPSE approaches that *use* neural networks as faster surrogates for the real model are part of this survey, we explicitly exclude works about exploring hyperparameters to *train* neural networks. We believe that the space of visualization and machine learning is sufficiently covered already [ERT*17, HKPC19, CMJ*20, CMJK20, YCY*20, SEG*21].

During our literature search, we found other surveys that mention papers we include here. These surveys do not necessarily relate to VPSE as a whole but could be helpful for VPSE designers working in specific application domains. Krispel et al. [KSU15] surveyed algorithmic shapes, that is, parametric representations of shapes. These can be valuable for visualization designers to find a multivariate representation of some spatial subspace, if necessary. Techniques of procedural modelling [STBB14] may also be useful in this regard. Sönmez brings the two together in a review of the use of examples for automating design tasks [Sön18]. The end goal of some papers in our survey was to produce a tangible object with some desired properties. This is called “functional fabrication,” of which Sá et al. [eSREPC16] provided a survey. While they focus on digital fabrication technologies, surveys exist for 3D printing [GZR*15], and visualization in smart manufacturing [ZLL*19] specifically. Simulations and how visualization, for example, with VPSE, may facilitate their use, were covered in a state of the art report about visual computing in materials science [HS17].

## 4 Method

**Figure 3:**
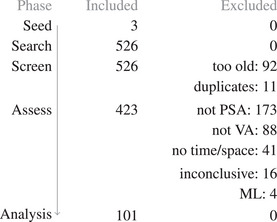
Flow diagram outlining our collection process.

This section describes our method for obtaining and processing surveyed papers. The objective of our survey is to understand how interactive visualizations and specially designed user interfaces support VPSE. To this end, we performed a systematic literature search to identify relevant papers. The process is depicted in Figure 3. We did a thorough keyword search with combinations and variations of “parameter”, “space”, “analysis”, “interactive”, “visual” and “exploration,” but were unsatisfied with the results as many relevant papers do not label themselves as such.

For this reason, we took a different approach. Our pool of relevant papers started with three seed papers: Another survey of visual parameter analysis [SHB*14], a survey of data processing pipelines [vLFR17], and a popular example of visual parameter analysis [TSM*11]. We expected that related works would be highly likely to cite at least one of them. We performed a snowball search from these seed papers, that is, looked at contained references. We added each suitable paper to the pool. The process was repeated for each paper in the pool until we did not find new papers. Then, in another pass through the pool, we repeated this in the forward direction, that is, looked for papers citing papers in our pool. We did this through Google Scholar. We then carried out the screening and assessment phase as outlined below. A list of all excluded papers together with exclusion reason is available as supplemental material.

### Inclusion Criteria

We included papers presenting interactive visualizations that facilitate exploring the parameter space of an input/output model as shown in Figure 1. Parameters and/or output had to exhibit spatial or temporal characteristics as outlined in Section 2. We did not restrict ourselves to a specific kind of model, for example, simulations, and used a broad interpretation of the term: If it could be interpreted as some function mapping some input onto some output, we considered the manuscript. Papers had to be published in 2010 or later in a peer‐reviewed outlet. Only papers written in English were considered. The paper's full text had to be available online in the form of an author's preprint or a research database accessible through the TU Wien library, such as IEEExplore or the ACM digital library.

### Exclusion Criteria

We excluded papers that did not fit in the above definitions. For example, fully automatic approaches and static visualizations were excluded, as were papers about interactive approaches without visualizations or a “human in the loop.” We excluded works where both parameter and output were abstract data. Our focus on physical space excludes spatializations, like clusterings or dimension reduction scatterplots, from our survey. An abstract parametric representation of a physical space was *not* an exclusion criterion. We excluded papers that were about exploring hyperparameters for a machine learning model, as we felt this space is already well covered by recent surveys (cf. Section 3). However, we included papers that used neural networks as a surrogate for the “real” model.

### Screening Strategy

The first author first checked the publication date, which excluded 92 out of 526 papers from our survey. Some papers were considered duplicates of others, for example, a conference paper that was later extended to a journal paper. These were removed as well (11 papers).

### Assessment Strategy

The first author read, in order, title, abstract, and conclusion. Then he looked at figures. Afterward, he searched for variations of “parameter” in the paper or for the reference that brought him to it. The first author read the paragraphs in question. Finally, he read the whole paper. He decided to include or exclude the paper at any point in the described sequence. He excluded 173 of the remaining papers that did not focus on parameter space analysis tasks or could not be interpreted as an input/output model. In addition, he excluded 88 papers because they did not describe visual‐interactive systems. The first author also excluded 41 papers that otherwise fit the topic but input and output were abstract data. In 16 cases, he could not determine the fit of a paper, so these also were excluded. Finally, he excluded four papers on training neural networks (i.e., the “parameters” were training hyperparameters).

**Figure 4:**
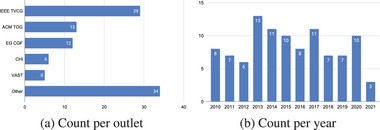
Statistics of the surveyed papers.

In the end we obtained 101 papers from 35 journals and conferences (Figure 4). The papers are listed in several tables in this survey (e.g., Table 1) and available as a SurVis installation online. Outlets are mostly from the broader visualization community, but include also others, like ocean engineering, space weather, or bioinformatics.

### Reflexive Thematic Analysis

With 57 papers from the final pool, we performed reflexive thematic analysis (RTA) [BC06, BC19, BC21a, BC21b]. RTA is a method to develop themes from qualitative datasets, such as interviews, videos, or research papers. In contrast to codebook or coding reliability approaches, RTA embraces that the researcher *develops* themes from the dataset and that they do not exist independently. Thus, the result is subjective, and we do not claim that our themes are consistent with what other people would develop. RTA [BC06, BC19] devises quality control steps in its process, which we followed. Specifically, phases 4 and 5 in the proposed process [BC06] require the researcher to review and further define themes. The goal is that “data within themes [cohere] together meaningfully, while there [are] clear and identifiable distinctions between themes.” Extensive discussions with co‐authors were part of these phases. For example, we considered structuring themes along space/time characteristics or parameter space analysis tasks. However, we deemed these efforts not fruitful as it was difficult for us to find coherent threads. Additionally, combining those potential dimensions yielded a too‐sparse matrix (compare Table 6 in Section 11). Consequently, we chose themes that underlie all parameter space tasks and all data characteristics. Codes were formulated by the first author based on described visualizations and available interactions with the system, as far as descriptions in the manuscript (textual and illustrative) and videos on the internet allowed it. For Input/Output Visualization themes (Section 6), the first author started with themes relating to existing concepts suggested in the literature, but these were expanded in the process. No pre‐existing themes were used for the other themes. The final codebook with extracts and a list of the included papers in RTA are available as supplemental material. The remaining 44 papers were used as a “test set,” like in a machine learning context, to verify the applicability of developed themes. As our themes are rather general (cf. Section 10), we encountered no issues in that process.

**Table 1:** Surveyed literature by supported parameter space analysis task [SHB*14]. Rows and columns are ordered by number of papers. A filled dot • means the task is supported, an empty dot ○ means it is not.

#		Optimization	Sensitivity	Uncertainty	Partitioning	Outliers	Fitting
25	[AME11, MSL*11, UKIG11, UIM12, BYMW13, LHH*13, Mar13, MDG*13, PWLS13, SK13, KWS*14, WKS*14, DLC*15, OAH15, ZSN*15, KGC*17, SMS*17, WMCM17, GPD*18, LDM*18, KGS19, DTSO20, KSG20, BHU*21, LDT*21]	•	○	○	○	○	○
14	[KP10, WFR*10, WDR11, CKGF13, vLBK*13, KSI14, UKSI14, DFL*15, PZR15, SOL*16, SXZ*17, YDH*17, DAM*19, MW20]	•	•	○	○	○	○
9	[GMG10, SSW*12, SRS*13, PDW*14, BBGS16, HG18, OKB*19, EST20, HWG*20]	○	•	○	○	○	○
6	[FMH16, OBJ16, SJJ*17, RGG18, JOR*19, RPI19]	○	•	○	•	○	○
4	[RWG*12, RBU*14, RLK*15, RSG21]	○	•	•	○	○	○
4	[AHRG10, MHG10, RWF*13, RBV*14]	•	•	○	○	•	○
4	[YCHK27, WAG*16, CKS*17, MGB*18]	•	•	○	•	○	○
3	[BHGK14, GT16, SJS20]	○	•	○	•	•	○
3	[BM10, SWG*18, WSL*20]	•	○	○	•	○	○
3	[BRG*12, BAF*13, BBGM17]	•	○	•	○	○	○
3	[MGSH10, MGJ*10, AE20]	•	•	○	•	•	○
3	[TSM*11, LRHS14, LRB*15]	•	•	•	○	•	•
2	[BDB*16, BHR*19]	○	○	•	○	○	○
2	[MAJH17, WLSL17]	○	•	•	○	○	•
2	[RCM*16, BBB*18]	○	•	•	•	○	○
2	[CLEK13, MBPdK13]	•	○	○	○	○	•
1	[SAJ*19]	○	○	○	○	•	○
1	[SWO*20]	○	○	○	•	○	○
1	[BPM*15]	○	○	•	○	○	•
1	[BLLS17]	○	•	•	○	•	○
1	[USKD12]	○	•	•	•	•	•
1	[PBCR11]	•	○	○	•	○	•
1	[BAF*14]	•	○	•	○	○	•
1	[HJM*11]	•	○	•	○	•	•
1	[HLW*20]	•	•	•	○	○	○
1	[MGS*14]	•	•	•	○	○	•
1	[BWMM15]	•	•	•	○	•	○
1	[LRE*12]	•	•	•	•	•	○
101	∑	68	60	25	25	19	13

## 5 Finding Parameter Settings

This section discusses interactions that lead to new (i.e., not previously analysed) parameter settings and outputs added to the underlying data table. We distinguish broadly how those parameter settings are obtained: Manually, either constrained to a particular parameter subspace or not, and automatically, either supervised or unsupervised. The classification of individual papers is listed in Table 2.

**Table 2:** Tabular overview of the Finding Parameter Settings theme. Rows and columns are ordered by number of papers. A filled dot • means the theme applies, an empty dot ○ means it does not. 30 papers were omitted, where the available input/output pairs are fixed and no new inputs can be obtained within the proposed system.

		Manual		Automatic	
#		Unconstrained	Constrained	Unsupervised	Supervised
20	[GMG10, WFR*10, AME11, HJM*11, UKIG11, WDR11, BAF*13, MBPdK13, SK13, BAF*14, RBU*14, BWMM15, BDB*16, BBGM17, SJJ*17, SXZ*17, YDH*17, OKB*19, HWG*20, MW20]	•	○	○	○
11	[AHRG10, MGJ*10, PBCR11, TSM*11, SSW*12, BHGK14, FMH16, WAG*16, HG18, RGG18, SAJ*19]	○	○	•	○
10	[MSL*11, UIM12, KSI14, UKSI14, OAH15, GPD*18, DAM*19, DTSO20, WSL*20, LDT*21]	•	•	○	•
7	[Mar13, KWS*14, WKS*14, DFL*15, SWG*18, SWO*20, BHU*21]	○	○	○	•
6	[BRG*12, RWG*12, vLBK*13, ZSN*15, GT16, WMCM17]	•	○	•	○
4	[BYMW13, PWLS13, DLC*15, LDM*18]	○	•	○	•
4	[LHH*13, RCM*16, SMS*17, HLW*20]	•	○	○	•
3	[CKGF13, KGS19, KSG20]	○	•	○	○
3	[KP10, CLEK13, YCHK27]	•	•	○	○
1	[MGS*14]	○	○	•	•
1	[BBGS16]	○	•	•	○
1	[KGC*17]	•	○	•	•
71	∑	44	21	20	27

**Figure 5:**
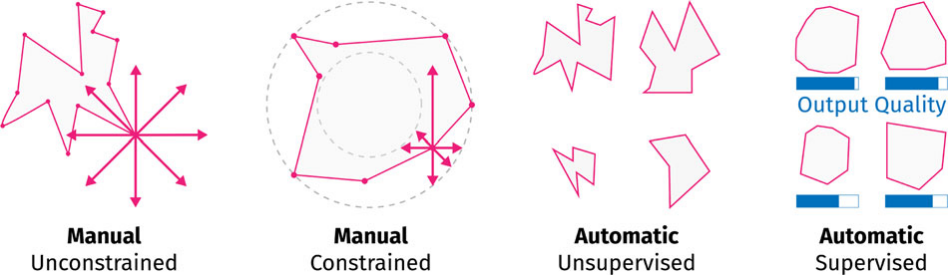
Sub‐themes of Finding Parameter Settings illustrated on a polygon (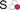 parameter). The two left images contain manual approaches, while automatic approaches are in the right two. Other than their counterparts, unconstrained and unsupervised approaches do not limit which parameter settings may be obtained.

Figure 5 shows a 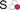 parameter (polygon) and illustrates the Finding Parameter Settings sub‐themes. We can imagine an algorithm that evaluates the roundness of the shape as our model. With Manual/Unconstrained, the parameter may be edited at will, thus taking any setting. As a result, any shape is possible. With Manual/Constrained, the parameter is restricted to a subspace, in this case, a ring: The currently edited vertex may be moved anywhere inside the subspace. Automatic techniques obtain parameter settings without or with little user interaction. Unsupervised approaches, like random sampling, traverse the parameter space independent of the output. Consequently, they may obtain very un‐round shapes. On the other hand, output quality (roundness) guides supervised approaches' parameter space traversal. In our example, they may, for example, only visit convex shapes. Regarding parameter space analysis tasks, we find that Manual/Constrained and Automatic/Supervised are commonly used to support *optimization* tasks, while the other two sub‐themes do not have a clear preference.

##### Surrogate Models

They may be useful in this endeavour as they can trade accuracy for decreased execution time. Some may be invertible, that is, it is possible to compute the input that produces a desired output. Autoencoder neural networks may be used to achieve that [HLW*20]. A special case is the work by He et al. [HWG*20], where the neural network learned directly the visualization image from the parameters. Simpler forms of regression were used as well, like linear models [MGS*14, BWMM15, MAJH17] or gaussian process models [TSM*11]. In some works the surrogate model was a learned function from human ratings capturing semantic attributes [YCHK27, KSI14, DAM*19]. However, it was often necessary to develop domain‐specific custom surrogates when it came to physical models [WDR11, UIM12, PWLS13, UKSI14, SMS*17].

##### Connection to Navigation Strategies

Sedlmair et al. [SHB*14] identified four navigation strategies in parameters spaces: Informed trial and error, local‐to‐global, global‐to‐local, and steering. While it may seem that there is overlap to our identified sub‐themes, for example, informed trial and error usually involves unconstrained manual input of parameter settings, they take different angles. The navigation strategies describe *how* an analyst *traverses* the space from one interesting parameter setting to the next. On the other hand, our themes describe *who identifies* the interesting settings in the first place and how computers and analysts work together to this end. Hence, they are independent of each other. For example, both local‐to‐global and global‐to‐local navigation depends on precomputed parameter settings. Due to the required number, these are usually obtained by automatic search methods but could also have been provided by domain experts [SOL*16]. Similarly, steering is about influencing the simulation while it happens. It is left open how and which effect should take place. In World Lines [WFR*10], this is completely left to the analyst (manual/unconstrained). However, as an automatic search was later introduced to this problem domain [WKS*14], it is conceivable that the system could recommend actions to the analyst at any point. Therefore, while navigation strategies may favor one parameter identification approach, we argue that the navigation strategy is mainly unrelated to who (human/computer) is responsible for finding interesting parameter settings.

### 5.1 Manual/Unconstrained

We classified papers to support unconstrained manual input when the user can enter any parameter setting supported by the model. Some systems restrict the available parameter space to make interactive computations possible, in which case they use a surrogate model that trades generality for execution speed. For example, in the context of clothing [UKIG11], the system does not allow all possible ways to stitch a garment, and in the context of architecture [SMS*17], the system only supports rooms and beams with certain angles. Because the surrogate model is still very much applicable for many use cases, we classify such papers as unconstrained.

**Figure 6:**
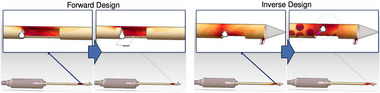
Forward and inverse design with direct manipulation of a canule (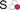 parameter); stress on surface (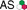 output) is shown embedded (Section 6.3) to the design. [CLEK13] © 2013 IEEE

Regarding how manual interactions with parameter spaces work, we can distinguish between indirect and direct manipulation. Direct manipulation, as defined by Shneiderman [Shn83], is characterized by (i) continuous representations of objects of interest, (ii) physical actions instead of textual commands, and (iii) rapid, incremental, and reversible actions. An example of direct manipulation of an abstract parameter can be found in interactive PCPs [MW20], while indirect manipulation would constitute every input method using form controls [RBU*14]. Direct manipulation of a 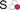 parameter would be to directly edit the spatial representation, for example, by growing/shrinking parts of a biopsy device with drag and drop (Figure 6, [CLEK13]). Indirect manipulation of such a parameter may happen through sliders for a parametric representation of it [SXZ*17]. While it is widely agreed that direct manipulation is superior to indirect manipulation, the latter can still be very effective if the system is interactive enough [KP10, HWG*20].

##### Indirect Manipulation

When applying this distinction it is visible that papers employing indirect manipulation commonly work with an abstract parameter space [SK13, KSI14, BWMM15, HLW*20]. A likely explanation is that there are no widely agreed‐upon visual representations of such parameter spaces. Other factors, such as openness to novel interfaces and existing preferences of domain experts participating in the respective design studies, probably play a role too.

##### Direct Manipulation

In the direct manipulation group, it is the other way around and parameters often have a temporal/spatial component. They are manipulated in any way that makes sense for the application domain: Wing shapes are drawn [UKSI14], as are walls [SMS*17] or shadows [LHH*13], time windows resized [BBGM17], and furniture is moved/rotated [MSL*11], through mouse operations on the visual representations. Novel input methods and modalities were explored sometimes, too. Kazi et al. [KGC*17] explored how generative modelling can be used to support the design stage. Within their system, DreamSketch, the designer sketches a design problem, such as a load‐bearing wall mount, using pen and tablet. The system then finds optimal solutions for varying combinations of design variables, which can be browsed within the sketch. Mohiuddin and Woodbury [MW20] explored a direct manipulation paradigm for a parametric representation of a 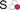 parameter (building design in architecture). They argue that, unlike with 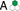 parameters in many works of this survey, “designers prefer direct engagement and manual exploration” over automated sampling. Hence, they propose novel interaction techniques for PCPs, such as sketching polylines, parallel editing, and quick generation of alternatives with operators, such as a cartesian product.

### 5.2 Manual/Constrained

Similar to the previous section, we classified papers as supporting constrained manual input when entering a parameter setting is still manual, but the system does not allow the user to enter or develop arbitrary parameter settings, even though the model would support them. The system often expects the available parameter subspace to lead to higher‐quality outputs. However, the restriction may also be a UI design decision to grapple with high‐dimensional parameter spaces. As with all input modes, this can be optional and in addition to other modes available in the system. We distinguish four approaches.

##### Restrict to Subspace

This approach occurs when the system allows free selection only in a continuous parameter subspace. Bao et al. [BYMW13] automatically identify subspaces of high‐quality solutions, allowing the user only to obtain combinations of those solutions. To do so, users select a point in a triangulated dimensionally‐reduced plot. The selected setting is found by barycentric interpolation. Kerr et al. [KP10] tested sliders against a gallery‐based system in a user study. The latter worked such that the user selects two dimensions and then sees a 5 × 5 gallery of the output with the two parameters step‐wise increasing/decreasing along the X or Y axis, respectively. The sliders were found to be superior because of more interactivity. Brunhart‐Lupo et al. [BBGS16] similarly restricted 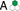 parameter selection to two dimensions with a “Parallel Planes” visualization in virtual reality.

##### Pick from Suggestions

With this approach, the system suggests discrete parameter settings, that is, points in the available subspace. These suggestions can be accepted, usually replacing the current setting. There are no further implications beyond that. Suggestions were used in interior design [MSL*11, WSL*20], shelf design [UIM12], image processing [KSI14], robot design [DAM*19], or graphic design [OAH15, DTSO20]. They were presented as a gallery.

##### Steer by Rating

**Figure 7:**
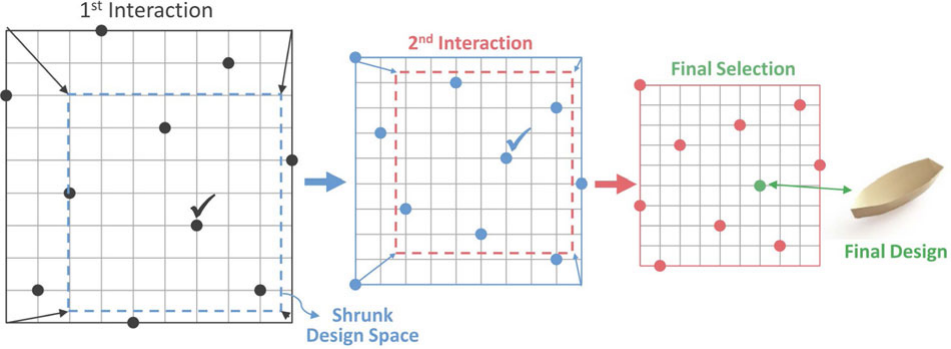
Steer by Rating: The parameter space is continually shrinked by selecting preferred solutions at its borders. [KGS19] © 2019 Pergamon

This approach works by shrinking the available subspace step by step until it is so small that it can be considered a point, that is, the desired solution. The shrinking process can take different forms. Koyama et al. proposed repeatedly searching along lines [KSSI17] and on planes [KSG20] via selection from galleries. Khan et al. (Figure 7, [KGS19]) uniformly sample the boundary of the available subspace and shrink it towards the selected direction, also via selection from a gallery. While the rating is binary (desired/undesired) in the previous examples, it is continuous in the case of probabilistic shape grammars [DLC*15]. The user rates outputs of such a grammar with a score of 0–100, and the system automatically modifies the grammar to produce preferred outputs more often.

##### Automatically Adapt Partial Solution

With this approach, the user provides the parameter subspace via a partial solution, and the system adapts it according to some objective. Liu et al. [LDT*21] recommend this strategy as part of their design guidelines for interactive optimization systems. Apart from their work, we found it in systems using sliders to select parameter settings, where the user may lock slider values and let the system automatically set free sliders [KSI14, YCHK27, DAM*19]. This approach may also be used with 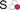 parameters. Umetani et al. [UKSI14] automatically set free design parameters of a model glider to maximize flight distance. Prévost et al. [PWLS13] automatically set free design parameters of a 3D model to balance it.

### 5.3 Automatic Search/Unsupervised

We classified papers as supporting unsupervised automatic search when they allowed automatically generating multiple parameter settings based on parameters alone. In other words, these techniques are not concerned with output quality or characteristics. In most cases, this comes down to sampling the parameter space by varying strategies within parameter ranges, possibly provided by the user through forms. This approach works best for abstract data. If a parameter is spatial or temporal, one could look for a suitable parametric representation [DFL*15, KGS19] so that standard techniques are applicable again. For independent scalars, one can draw from predefined distributions, for example, uniform or normal. Binning continuous variables, that is, defining a step size, was also a strategy. The step values can then be independently increased/decreased [KP10] to “walk” in desired directions or put together with a cartesian product (or “full factorial”) to obtain all possible combinations. Multiple dependent variables may be sampled with Latin hypercube sampling or low‐discrepancy sequences. A contour tree [DFL*15] or a clustering helps to prune too similar parameter settings, but post‐processing ventures into supervised methods, which we discuss in Section 5.4.

A few systems warrant dedicated discussion. Matković et al. [MGS*14] combine both approaches to automatic search. First, the parameter space is sampled coarsely and unsupervised. Then the analyst builds a linear regression model based on desirable simulation outputs. The system then uses this surrogate model to identify relevant parameter subspaces (supervised search). Ribičić et al. [RWG*12] sample from a normal distribution when sketching uncertainty into spatio‐temporal flood simulations, like an uncertain breach location. Torsney‐Weir et al. [TSM*11] sample from an 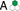 parameter space with a time budget and afterward quantify uncertainty and expected gain of parameter subspaces with a gaussian process model.

### 5.4 Automatic Search/Supervised

On the other hand, we classified systems as supporting supervised automatic search when they could automatically identify at least one parameter setting while simultaneously considering output quality or characteristics. This approach was a prerequisite for, or more generally part of, many papers offering a constrained manual search (Section 5.2). We distinguish papers based on the optimization method used.

##### Unsupervised Search With Post‐Processing

In this approach, the parameter space is first sampled in an unsupervised fashion (cf. Section 5.3), and acquired outputs are then post‐processed to remove undesired outputs from the result set. More specifically, this entailed removing everything but the top‐k results [KSI14, DAM*19, WSL*20], everything that does not satisfy a property or constraint [RCM*16, SWO*20], or too similar parameter settings [DFL*15]. This strategy can work for low‐ to medium‐dimensional 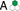 parameter spaces. For example, Swearngin et al. [SWO*20] used branch‐and‐bound (search) and a constraint solver (post‐processing) successfully for a layout of six interface elements, which amounts to a 24‐dimensional parametric representation of these spatial objects.

##### Exact Optimization Methods

The authors found a domain‐specific optimization formula for their problem in several surveyed works. They used existing exact‐solving techniques, such as Mixed Integer Linear Programming, Quadratic Programming, or Gradient Descent. The problem domains include vehicle routing [LDT*21], brachytherapy [LDM*18], grid‐based layout design [DTSO20], robot design [GPD*18], balancing 3D models [PWLS13], designing air gliders [UKSI14] or architecture [SMS*17]. The technical details of the optimizer are usually hidden behind the user interface as much as possible. Liu et al. [LDT*21] provide design guidelines for such interactive optimization scenarios.

##### Metaheuristics

This optimization technique is used for functions that behave in ways that make the former category (exact optimization methods) inapplicable. Marsault [Mar13] used an interactive evolutionary algorithm framework to obtain possible building designs, where the fitness (objective) function includes terms such as compactness or casting few shadows on neighbouring buildings. Berseth et al. [BHU*21] use Covariance Matrix Adaption to optimize a single floor plan for metrics of space syntax. To this end, the user provides 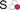 parameter ranges, for example, how much a wall segment may be moved or rotated, and is afterward presented with a gallery of possible solutions.

##### Custom/Other Methods

**Figure 8:**
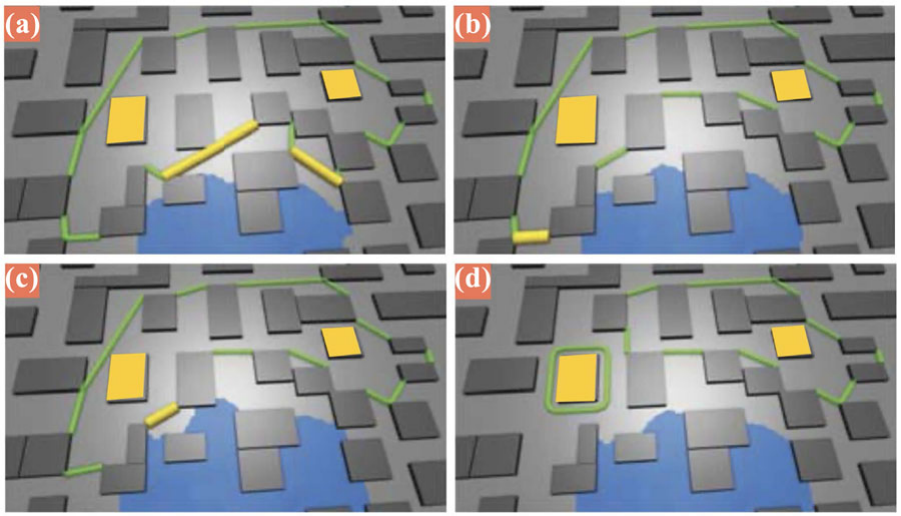
Supervised automatic search for flood barrier settings (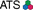 parameter). The perimeter is continually shrinked (a–d) when water touches it. [KWS*14] © 2014 IEEE

Authors of surveyed papers also used custom solvers or other optimization techniques than in the categories above, such as Markov Chain Monte Carlo sampling [MSL*11], topology optimization [KGC*17], or autoencoders with Activation Maximization [HLW*20]. It is notable that the parameter of all papers in this category [MSL*11, UIM12, LHH*13, KWS*14, WKS*14, KGC*17, SWG*18, HLW*20] has a spatial component (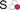, 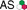, 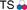, or 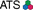). As a domain‐specific example, Konev et al. (Figure 8, [KWS*14]) implemented an automatic search for flood barrier settings (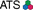). A perimeter should protect critical infrastructure. The perimeter is adjusted when water touches or passes over it, for example, by changing the barrier type or moving the perimeter back.

##### Multiple Methods

Some papers did not fit in one of the above‐mentioned categories because they employ multiple optimization methods. O'Donovan et al. [OAH15] use the MCMC approach by Merrell et al. [MSL*11] to obtain high‐quality layout designs and use Nonlinear Inverse Optimization to estimate parameters of their graphic design model. Bao et al. [BYMW13] find an initial set of building candidates by a metaheuristic (Simulated Annealing), and each candidate is further optimized with Quadratic Programming. Dang et al. [DLC*15] use various methods to simplify the definition of probability density functions for shape grammars, including, for example, Conjugate Gradient for feature relevance detection.

## 6 Input/Output Visualization

Table 1 lists papers in our survey based on supported parameter space analysis tasks. It can be seen that several tasks, including the two most frequent ones, ask about a relation between parameters and output. For instance, *optimization* is about finding a parameter setting that produces a “best” output according to some objective, and *sensitivity* asks how much change in output one can expect when varying a given parameter. Thus, an important high‐level goal in VPSE is to reconcile and compare the parameter and output spaces of the model. This theme explores how this task can be supported with visualizations.

**Table 3:** Visualization strategies concerning inputs and output in our survey. Rows and columns are ordered by number of manuscripts. A filled dot • means the theme applies, an empty dot ○ means it does not. 12 papers not using composite input/output visualizations were omitted.

#		Juxtaposition	Embedding	Superposition	Alignment	Seq. Superpos.	Overloading	Expl. Encoding	Integration	Nesting
22	[BM10, MGSH10, MGJ*10, PBCR11, Mar13, MDG*13, MGS*14, PDW*14, BWMM15, LRB*15, ZSN*15, OBJ16, RCM*16, CKS*17, MAJH17, WLSL17, WMCM17, OKB*19, RPI19, AE20, DTSO20, SWO*20]	•	○	○	○	○	○	○	○	○
9	[LRE*12, LRHS14, RBU*14, RLK*15, BDB*16, BBB*18, JOR*19, SAJ*19, KSG20]	○	○	○	•	○	○	○	○	○
9	[RWG*12, LHH*13, MBPdK13, KWS*14, BBGM17, KGC*17, LDM*18, WSL*20, LDT*21]	○	○	•	○	○	○	○	○	○
8	[AHRG10, TSM*11, SRS*13, vLBK*13, BPM*15, BBGS16, YDH*17, HG18]	○	•	○	○	○	○	○	○	○
5	[CLEK13, RWF*13, WKS*14, RGG18, SJS20]	○	•	•	○	○	○	○	○	○
4	[UKIG11, UKSI14, SWG*18, HWG*20]	○	○	○	○	•	○	○	○	○
4	[RBV*14, DFL*15, MGB*18, MW20]	•	•	○	○	○	○	○	○	○
3	[UIM12, CKGF13, RSG21]	○	○	○	○	○	•	○	○	○
3	[KP10, HJM*11, KSI14]	○	○	○	•	•	○	○	○	○
3	[SSW*12, GT16, SJJ*17]	○	○	•	•	○	○	○	○	○
2	[AME11, BHGK14]	○	•	○	○	○	•	○	○	○
2	[GMG10, SXZ*17]	○	•	○	○	•	○	○	○	○
1	[YCHK27]	○	○	○	○	•	○	•	○	○
1	[EST20]	○	○	○	•	○	○	○	○	•
1	[MHG10]	○	○	•	○	○	•	○	○	○
1	[DAM*19]	○	○	•	○	•	○	○	○	○
1	[WFR*10]	○	•	○	○	○	○	○	○	•
1	[USKD12]	○	•	○	○	○	○	○	•	○
1	[WAG*16]	○	•	○	○	○	•	•	○	○
1	[BRG*12]	○	•	•	•	○	○	○	○	○
1	[BHR*19]	○	•	•	•	○	○	○	•	○
1	[BAF*13]	•	○	○	○	○	○	○	•	○
1	[WDR11]	•	○	○	○	•	○	○	○	○
1	[PZR15]	•	○	○	•	○	○	○	○	○
1	[HLW*20]	•	○	•	○	○	○	○	○	○
1	[BAF*14]	•	○	•	○	○	○	○	•	○
1	[FMH16]	•	•	○	○	○	•	○	○	○
89	∑	32	27	23	19	12	8	4	2	2

Notably, we considered in sub‐themes only visualizations that involve both the model's input (static or dynamic) and output. Hence, for example, a Superposition of multiple 3D shapes that are all the output of a shape generator would not be considered in this section. We do not focus solely on dynamic inputs (parameters) in this section, as the output of some models (e.g., time series processing) is a modified version of a static input. Thus the relation between outputs and static inputs is also necessary for parameter space analysis in that context.

**Figure 9:**
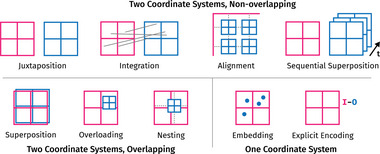
Sub‐themes of Input/Output Visualization. The grids refer to coordinate systems of visualizations, where red is generally the input and blue the output.

**Figure 10:**
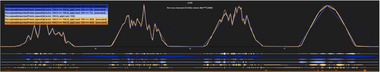
Example for combination of Input/Output Visualization sub‐themes on 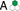 parameter, 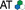 static input and 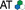 output: Superposition, Embedding, Alignment, Explicit Encoding. [BHR*19] © 2019 Wiley

In contrast to other themes, we started with a pre‐made set of initial sub‐themes. As parameter and output spaces often have different characteristics (dimensionality, space, time), we expected to see composite visualization approaches. Therefore, we took initial sub‐themes from Javed & Elmqvist's [JE12] composite visualization taxonomy (Juxtapose, Integration, Overloading, Nesting, Superimpose). In addition, we added Explicit Encoding from the visual comparison taxonomy by Gleicher et al. [GAW*11], as we also expected detailed comparisons within or between inputs and outputs to be necessary for some situations. In the coding process, we found these themes insufficient and extended them by Embedding, Alignment, and Sequential Superposition. The themes differentiate by how many visualization coordinate systems there are (one or two) and whether or not these occupy the same display area. We illustrate them in Figure 9. As the themes describe rather high‐level approaches to composing multiple visualizations, they may also be combined. For instance, Bernard et al. (Figure 10) discuss the impact of a time series processing algorithm on the input time series. In that image, we see the themes Superposition (input and processed time series), Embedding (color mapping of a parameter value), Explicit Encoding (difference between input and processed time series in ribbons below), and Alignment (by time axis on the horizontal and by parameter value on the vertical axis in the ribbons). Hence, our proposed themes, building on previously suggested approaches, form a design space for composite visualizations.

### 6.1 Juxtaposition

**Figure 11:**
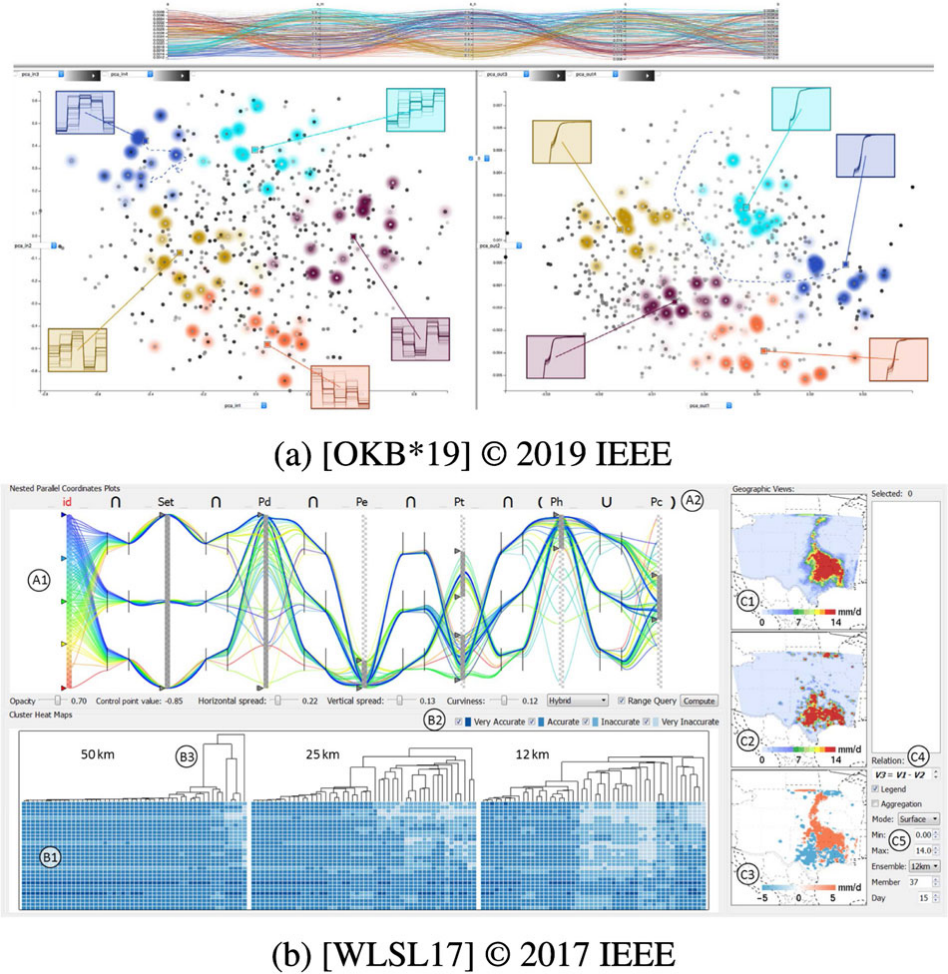
Examples for Juxtaposition (Section 6.1). (a) Dimensionally‐reduced views of a 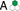 parameter (left) and a time series (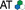 output, right) support *sensitivity* analysis. (b) Coordinated multiple views showing 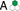 parameters (top) and accuracy (bottom, right) of a precipitation forecasting model (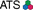 outputs) support *uncertainty* analysis.

Juxtaposition (Figure 11) refers to separate input and output visualizations, which are shown side‐by‐side, and any layout to do so is possible [JE12]. Juxtaposition is a popular strategy in our survey. It allows specialized visualizations of the respective data type, for example, a parallel coordinates plot for the 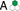 parameter and a gallery of the resulting 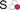 3D models [AE20], or 2D embeddings of 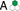 parameters and 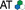 time series (Figure 11a, [OKB*19]). Involved views are often conceptually linked through the Gestalt principles of common fate (when the analyst manipulates one view, the other changes immediately as well), or similarity (selected data cases highlighted in the same fashion everywhere). Because respective visualizations can be positioned anywhere and little shared visual cues are necessary, this strategy is flexible and can be applied to any data type combination.

Given the popularity of Juxtaposition it is unsurprising that it was used to support diverse parameter space analysis tasks. Figure 11a shows a 2D projection scatterplot of 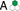 parameters on the left, next to a 2D projection scatterplot of time series (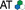 output). The analyst may select a subset of data cases in one scatterplot, thus highlighting connected data cases in the other. By comparing how much data cases spread and in which directions, it is possible to do *sensitivity* analysis.

Zaman et al. [ZSN*15] propose a user interface for a geometry generator, that is, the 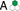 parameter is a graph of parameterized drawing operations, and the 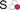 output is a vector image. Juxtaposing the graph editor and the output allows specialized visualizations for both. The desired vector image (*optimization* task) is created via indirect manipulation (Section 5.1).

Wang et al. [WLSL17] propose a Nested Parallel Coordinates Plot (NPCP) that depicts 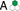 parameter settings and visualizations for spatial/temporal accuracy (derived feature from 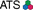 simulation output and measured observation) of the forecast underneath and to the right. Data cases can be interactively filtered in the NPCP, and the accuracy of the forecast in space and time is explored in the other views (*uncertainty*).

### 6.2 Superposition

**Figure 12:**
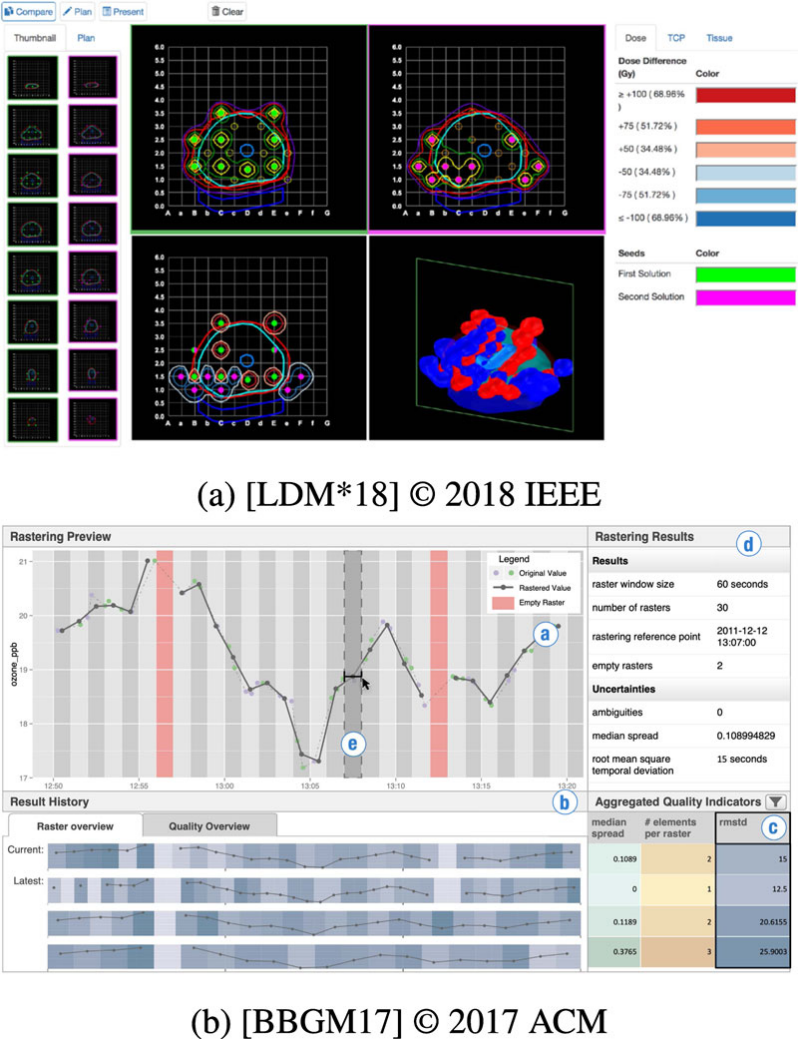
Examples for Superposition (Section 6.2) to support an *optimization* task. (a) Radiation seed positions (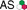 parameter), organs at risk (static 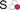 input) and radiation dose (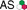 output) of brachytherapy plan shown on axis‐aligned slices (top row). (b) Original (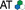 input) and rastered time series (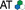 output) as well as raster size (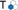 parameter) shown on common time axis in top left part of the layout.

Input and output visualizations are overlaid onto each other with Superposition (Figure 12): They occupy the same display area and share their coordinate system. While this allows detailed comparisons, the disadvantage of this strategy is that it only works with visual marks of the same domain, for example, lines depicting time series in the same interval or trajectories referencing the same geographical space.

Superposition was often used in works that support the analysis tasks *optimization* or *sensitivity*. As an example, to support *sensitivity* analysis, Desai et al. [DAM*19] superimpose regression lines onto the 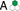 parameter sliders. The line indicates whether a target semantic attribute of the robot motion (e.g., “angry”), that is, the 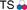 output, increases or decreases with the parameter. Because of the superposition, it is easy to see the impact of potential changes of the parameter on the output in detail.

In brachytherapy (Figure 12a), doctors place radiation seeds, which are injected into the patient's body to control tumours, on a matrix grid. By superpositioning seed amount and location (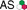 parameter), organs at risk (static 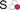 input), and radiation dose (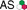 output), doctors can *optimize* radiation dose.

Another example is a flood simulation, where the analyst defines location and severity of a sewer overflow (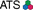 parameter) within the 3D scene. As it is otherwise difficult to appreciate a given emission rate, the flood simulation is run immediately, and the simulation output is previewed within the region of interest. In such a scenario, the main goal is often to protect core infrastructure. The amount of water visible at this stage can then already suggest whether or not this infrastructure is in danger (*uncertainty*).

With data mining or prediction algorithms that have both a temporal input and a temporal output, such as a time series smoothing operation, the goal is often to balance specific properties in the output with an overall resemblance to the input. For example, a moving average operation should smooth out the noise, but the general shape of the original time series should remain intact. This task is an *optimization* task, and superpositioning original and processed data points was helpful to achieve it [BHR*19, BBGM17, BAF*14, BRG*12], cf. Figure 12b.

### 6.3 Embedding

**Figure 13:**
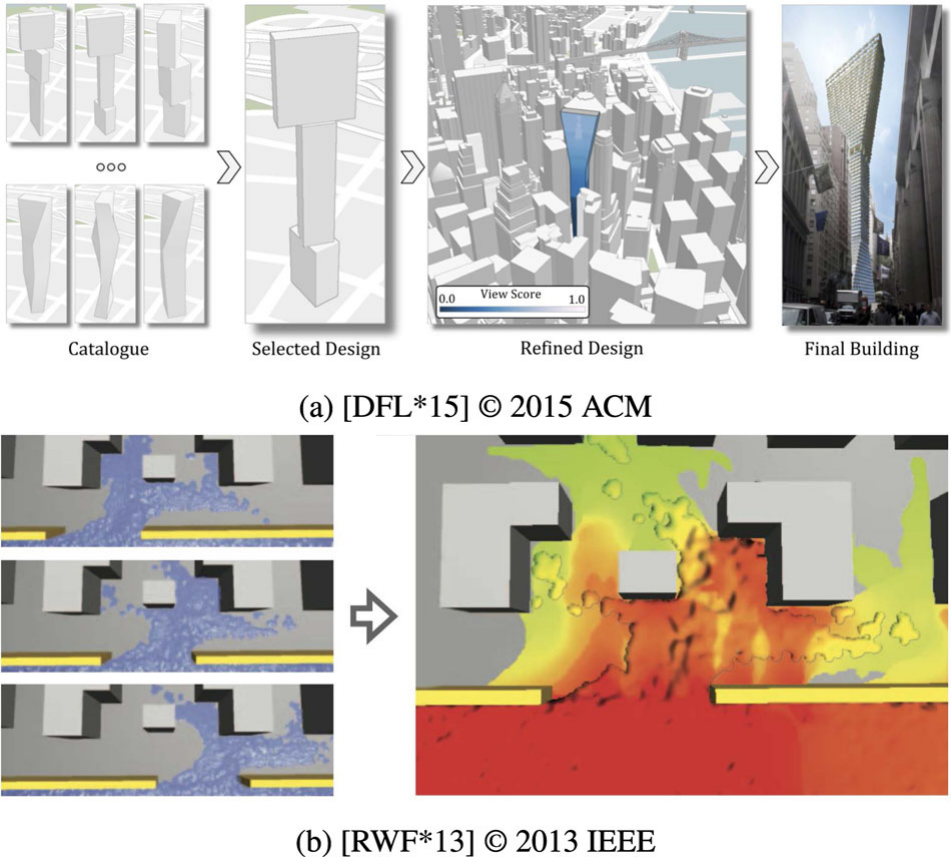
Examples for Embedding (Section 6.3). (a) View quality (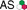 output) out of a skyscraper's (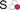 parameter) windows (“refined design”) to support *optimization*. (b) Aggregated water heights (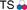 output) indicate *sensitivity* to breach location (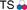 parameter, yellow horizontal lines).

We refer to Embedding (Figure 13) in the sense of “making an integral part of something” [Mer]. There is only one visualization and one coordinate system. Input and output are combined into the same visualization via mapping to visual channels. Hence, Embedding may technically be considered not a composition of two visualizations but rather the combination into one. Examples include scatterplots that show a parameter on one axis and a (possibly derived) output on the other [FMH16], parameters and outputs as axes in a parallel coordinates plot [SRS*13], colour‐coding output quality in a tilemap of two parameters [AHRG10] or on 3D shapes [DFL*15].

Depending on what part of the output is of interest and visualized, Embedding can support several parameter space analysis tasks. When the goal is to *optimize* some objective, this objective measure is often embedded into a visualization of the parameter. For example, Figure 13a shows a skyscraper (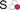 parameter). For investors, it is important to charge high rents for the apartments, which they can do when the views from the apartment are excellent, for example, when important landmarks are visible. This view quality is the 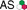 model output and is visualized with colour on the skyscraper's surface. The task of designing a financially viable apartment building is thus reduced to browsing building alternatives and comparing their color distribution. Similar examples can be found in manufacturing [CLEK13, SXZ*17] or 3D scanning [AHRG10].

Embedding can also support *sensitivity* analysis when parameter and output are combined into visualizations that are suited to this type, for example, scatterplots [MGB*18], parallel coordinates plots [SRS*13], or a combination of the latter with cobweb charts [RBV*14]. *Uncertainty* analysis can be carried out when multiple outputs are aggregated prior to Embedding. In the context of flood simulations, this was useful to visualize, for example, the highest water level associated with any 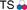 parameter setting (such as breach location) at any time step. From Figure 13b, it can be seen which areas are flooded or not (coloured or gray) and how badly (green‐red colourmap).

Finally, when the difference between model output and a reference value is encoded with Embedding [USKD12], the parameter space analysis task *fitting* is supported. Analysts can quickly find and select the parameter setting that closely matches physical measurements for further inspection.

### 6.4 Alignment

**Figure 14:**
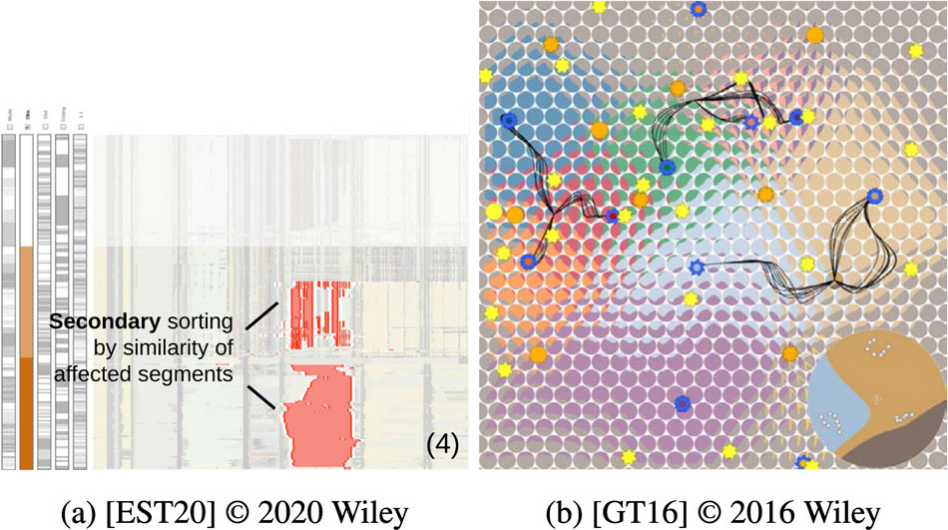
Examples for Alignment (Section 6.4). (a) Spreadsheet‐like visualization with 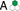 parameter on the left and 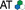 output on the right shows output *sensitivity* to parameter settings. (b) Particle trajectory glyphs (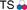 output) are aligned in a grid pattern according to initial position of the particle (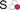 parameter), thus supporting *partitioning*.

Alignment (Figure 14) refers to situations where inputs and outputs are visualized in separate visualizations. Hence their visualizations' coordinate systems are separate and do not overlap. In contrast to Juxtaposition and Integration, the visualizations cannot be rearranged at will. Examples of Alignment include spreadsheet‐like visualizations (data for a row is horizontally aligned, Figure 14a) or grid‐like visualizations (Figure 14b). Visualizations in the Alignment theme have similarities to pixel‐oriented visualizations [Kei00] in that the individual visualizations can be, but are not necessarily, quite simple. The image that emerges by aligning many of those visualizations is more than the sum of its parts.

We found Alignment to support diverse parameter space analysis tasks. When temporal outputs are sorted vertically by parameter settings (Figure 14a), dependencies and correlations between parameter settings and output can be highlighted (*sensitivity*). Of course, the exact sorting order must be flexible and changeable by the analyst (cf. Section 7.4).

Alignment also facilitated *uncertainty* analysis in time series preprocessing (Figure 10). The amount of uncertainty (difference between original and output time series) introduced by different preprocessing settings was aligned underneath the original time series. The analyst can quickly gather from that which parameter setting leads to an output that is still truthful to the original time series.

Alignment was used in the same spirit as the previously mentioned pixel visualizations in the context of vector field topology (Figure 14b). The individual visualizations are circular glyphs in which colour encodes where particles end up. The initial velocity and direction of the particle are mapped to distance and angle in the glyph, while the position of the glyph encodes the initial position of the particle. When zooming out, all possible behaviours of particles become visible (*partitioning* task).

### 6.5 Sequential Superposition

**Figure 15:**
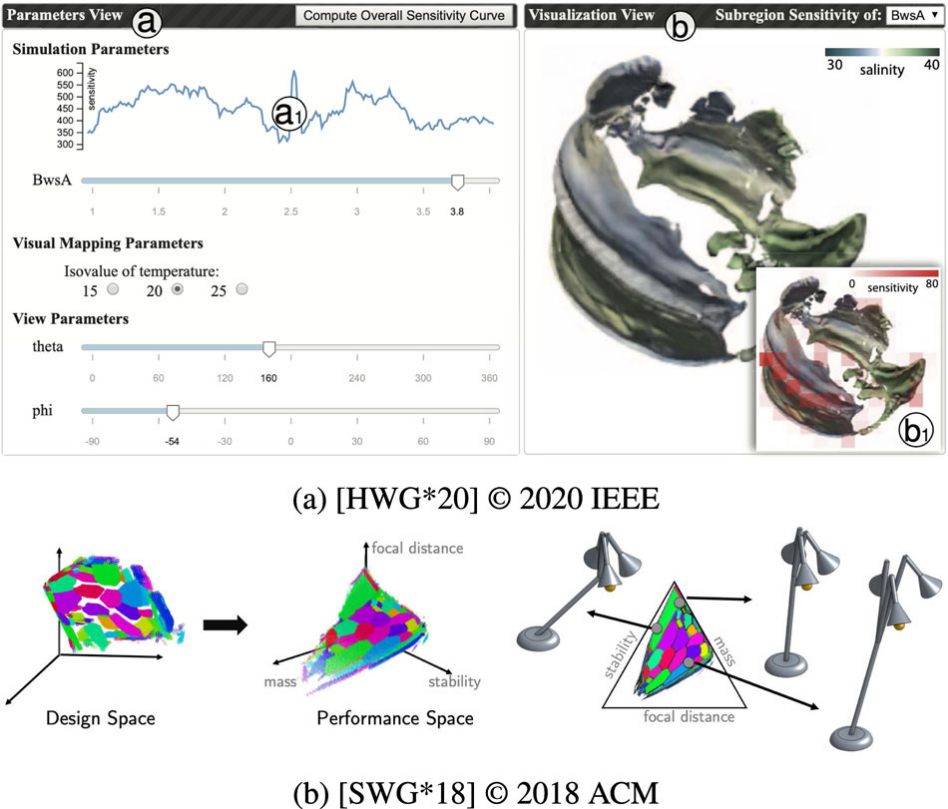
Examples for Sequential Superposition (Section 6.5) and *optimization*/*sensitivity* tasks. (a) 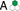 parameters (left) and volume visualization (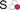 output, right) of an ocean simulation. (b) 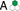 output space is divided into Pareto‐optimal sections, 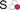 parameter setting (lamp designs) is shown to the side.

With Sequential Superposition (Figure 15), input and output visualizations have separate coordinate systems. They do not occupy the same display area, but the output visualization shows a single output that is rapidly exchanged over time after user interaction in the input visualization. While this theme could be seen as Juxtaposition (Section 6.1), we argue that the high level of interactivity makes this approach qualitatively different. The user controls the emerging movie, enabling trial and error, probing, and “what if” analysis. In other words, by quickly experimenting with varying parameter settings and observing the model output, VPSE becomes possible. The controls are very often juxtaposed sliders, but more sophisticated visualizations are possible [UKSI14, SWG*18].

Sequential Superposition enabled mainly *optimization* and *sensitivity* tasks. Rapid exploration of the output space allows for quickly finding relevant parameter subspaces, which can be further refined. On the other hand, the influence on the output can be determined by varying one parameter and observing the output while keeping other parameters fixed. He et al. [HWG*20] developed a surrogate model for a computationally expensive ocean simulation by training a neural network to produce the desired visualization image directly. As pictured in Figure 15a, analysts can freely change simulation, visual mapping, and view parameters on the left while the respective volume visualization is shown on the right. Another example in the same fashion, but without sliders, can be seen in the work by Umetani et al. [UKSI14], where a direct manipulation wing design interface is used instead.

Sequential Superposition can also work the other way around when the output space is explored, and parameters are observed. Figure 15b shows such a case. The triangle on the right depicts areas of Pareto‐optimal designs in the output space. Pareto‐optimality refers to the situation where no objective can be improved without another one worsening. When the user hovers over the triangle, possible 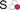 parameter settings (the lamps) leading to this Pareto‐optimum are shown on the side. This paradigm is sometimes referred to as “inverse design.”

### 6.6 Overloading

**Figure 16:**
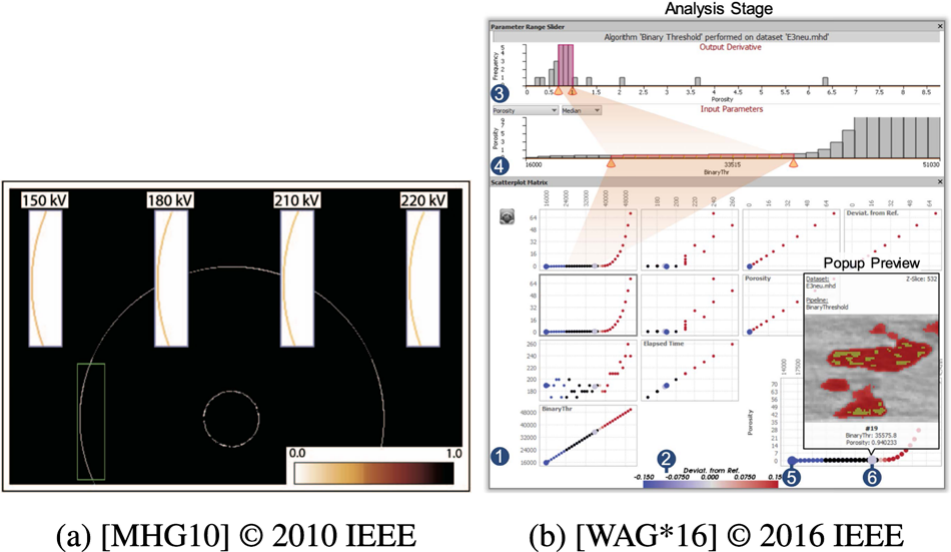
Examples for Overloading (a, Section 6.6) and Integration (b, Section 6.7). (a) Detected edges (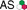 feature) in images scanned with 3D X‐ray computed tomography (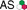 output) and different 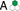 scan parameters (*optimization*, *sensitivity*). (b) Integration of 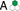 parameter and derived 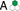 feature with trapezoids—comparing side lengths of the trapezoid enables *sensitivity* analysis.

With Overloading, input and output visualizations overlap in the display area, but their coordinate systems differ. The position of the overlaid coordinate system is irrelevant, that is, positions, distances, and sizes in one visualization do not directly translate to the other. An example is overlaying glyphs [RSG21]. While the space depicted in the overlaid graphics in Figure 16a is the same as in the selected region of interest underneath, the offset and repetition make the approach different from Superposition.

Raith et al. [RSG21] show *uncertainty* glyphs at locations where ocean eddies were detected (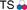 output). Glyphs depict whether the uncertainty source is time (number of time steps without Eddie), environmental conditions (e.g., Eddie detected only at certain water temperature), or the 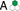 parameter setting of the detection algorithm.

Beham et al. (Figure 23a left, [BHGK14]) also used Overloading to overlay images of 3D models (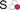 output) on the parallel coordinates plot (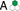 parameter space visualization), thus enabling *partioning*.

Malik et al. (Figure 16a), who show detected edges in scanned images (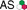 output), obtained various scanning configurations (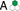 parameter). Seeing multiple of those in the same view enables both *optimization* when the analyst can pick the setting with the “best” edges, and *sensitivity*, as the analyst can investigate the impact of a few settings of one parameter on the detected edges in the selected region of interest.

### 6.7 Integration

Integration refers to Juxtaposition, that is, separate non‐overlapping input and output visualizations, but with explicit links between marks of the two visualizations [JE12]. Only Weissenböck et al. [WAG*16] and Yumer et al. [YCHK27] used this approach. In the former case (Figure 16b), a trapezoid connects the respective 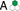 parameter and 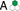 derived feature ranges of histograms. Thus, the trapezoidal annotation's shape hints at the *sensitivity* of the parameter. The integrating links connect 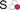 outputs to a point in the 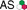 parameter space of the latter example, thus enabling *partitioning*.

### 6.8 Explicit Encoding

**Figure 17:**
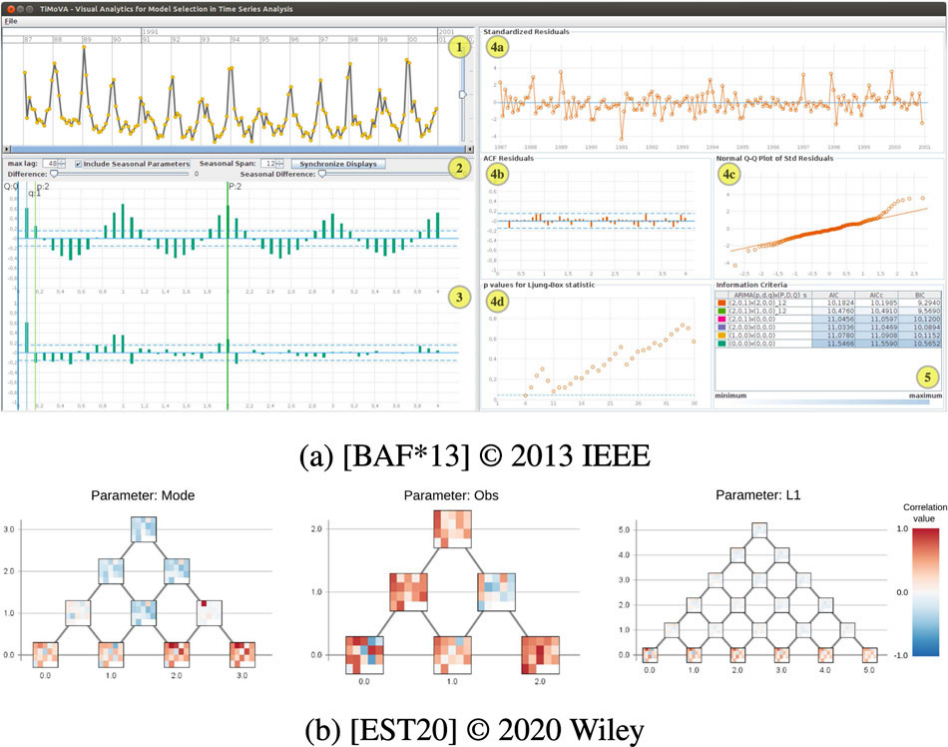
Examples for Explicit Encoding (a, Section 6.8) and Nesting (b, Section 6.9). (a) Residual plots (4a, 4b) utilize Explicit Encoding to show if any seasonal patterns persist between the original and modelled time series (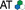), an *optimization* task in time series modelling. (b) Correlation to 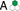 feature of 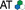 output (matrix) nested into visualization of 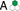 parameter value intervals (tree) showing *sensitivity* of parameter range to output feature.

Explicit Encoding refers to only one coordinate system and visualization showing the difference between inputs and outputs with the Explicit Encoding idiom [GAW*11]. As specialized comparison visualizations were not that common in the papers we surveyed, this category also remains somewhat small. Explicit Encoding was mostly used with time series processing, highlighting where original (input) and output time series differ (Figure 17a). In that context, the idiom usually supports an *optimization* task.

### 6.9 Nesting

Nesting means that input and output have separate visualizations and coordinate systems, they overlap in the display area, and the positioning of the overlaid coordinate system matters. The overlaid coordinate systems are nested into the marks of the “host” visualization. Hence, like Overloading, but position matters. Like Embedding, but marks are complete visualizations with their own coordinate system. Like Alignment, but there is a proper host visualization and not only imagined coordinate axes.

Working with time series segmentations, Eichner et al. [EST20] added small correlation matrices into the marks of a visualization depicting different 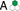 parameter ranges (Figure 17b). In doing so, it becomes visible which ranges of a given parameter influence which features in the output, for example, the number of segments with a particular label (*sensitivity* analysis).

## 7 Data Case Organization

In this section, we discuss in more detail the sub‐themes of the Data Case Organization theme (Table 4). Many systems work with multiple parameter/output pairs with temporal/spatial characteristics. A clear challenge to effective data analysis is the amount and complexity of the involved data. Hence, VPSE systems use varying strategies to reduce the amount and complexity of the data the analyst has to reason about. We found five strategies to achieve that (Figure 18), which, considering they resemble buildings blocks of an SQL SELECT statement, can be seen as basic querying operations. Their outcome may be visualized directly, or combined with each other to arrive at sophisticated concepts. For example, we could obtain the accuracy of a model in a given spatial region of the output by (i) filtering reference and output data to the spatial region (focusing), (ii) computing the difference between reference and outputs (derivation), (iii) computing the average of differences (aggregation). If this process is repeated for multiple regions, regions may be ranked (sorting) or clustered (grouping) by accuracy, thus supporting, for example, *uncertainty* analysis. Other important scalars obtained by combining these operations are sensitivity indices, of which several [Ham94, Bor07, GI12] exist.

**Table 4:** Overview of Data Organization theme. Rows and columns are ordered by number of papers. A filled dot • means the theme applies, an empty dot ○ means it does not. 29 papers where this theme does not apply were omitted.

#		Focusing	Derivation	Aggregation	Arrangement	Grouping
10	[AME11, MDG*13, SK13, DFL*15, ZSN*15, BBGS16, GT16, LDM*18, OKB*19, SWO*20]	•	○	○	○	○
10	[AHRG10, USKD12, SRS*13, RBV*14, BDB*16, SJJ*17, RGG18, JOR*19, RPI19, RSG21]	•	○	•	○	○
10	[BRG*12, CLEK13, BPM*15, BWMM15, KGC*17, MAJH17, WLSL17, YDH*17, BHR*19, WSL*20]	•	•	○	○	○
8	[WKS*14, DLC*15, LRB*15, PZR15, RLK*15, SOL*16, MGB*18, EST20]	•	•	○	•	○
8	[MGSH10, MGJ*10, TSM*11, MGS*14, WAG*16, CKS*17, SAJ*19, SJS20]	•	•	•	○	○
4	[RWF*13, BHGK14, FMH16, BLLS17]	•	•	•	○	•
3	[BYMW13, PDW*14, SWG*18]	○	○	○	○	•
3	[SSW*12, LRHS14, BBB*18]	○	○	○	•	○
3	[BAF*13, KGS19, MW20]	○	•	○	○	○
3	[BM10, PBCR11, HLW*20]	•	○	○	○	•
3	[OBJ16, WMCM17, AE20]	•	•	○	○	•
2	[vLBK*13, BBGM17]	○	•	•	○	○
2	[WFR*10, LDT*21]	•	○	○	•	○
2	[MHG10, LRE*12]	•	•	•	•	○
1	[RCM*16]	•	○	•	○	•
72	∑	58	38	29	17	16

**Figure 18:**
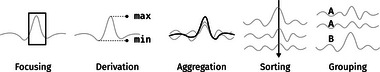
Sub‐themes of Data Case Organization illustrated on a time series.

### 7.1 Focusing

**Figure 19:**
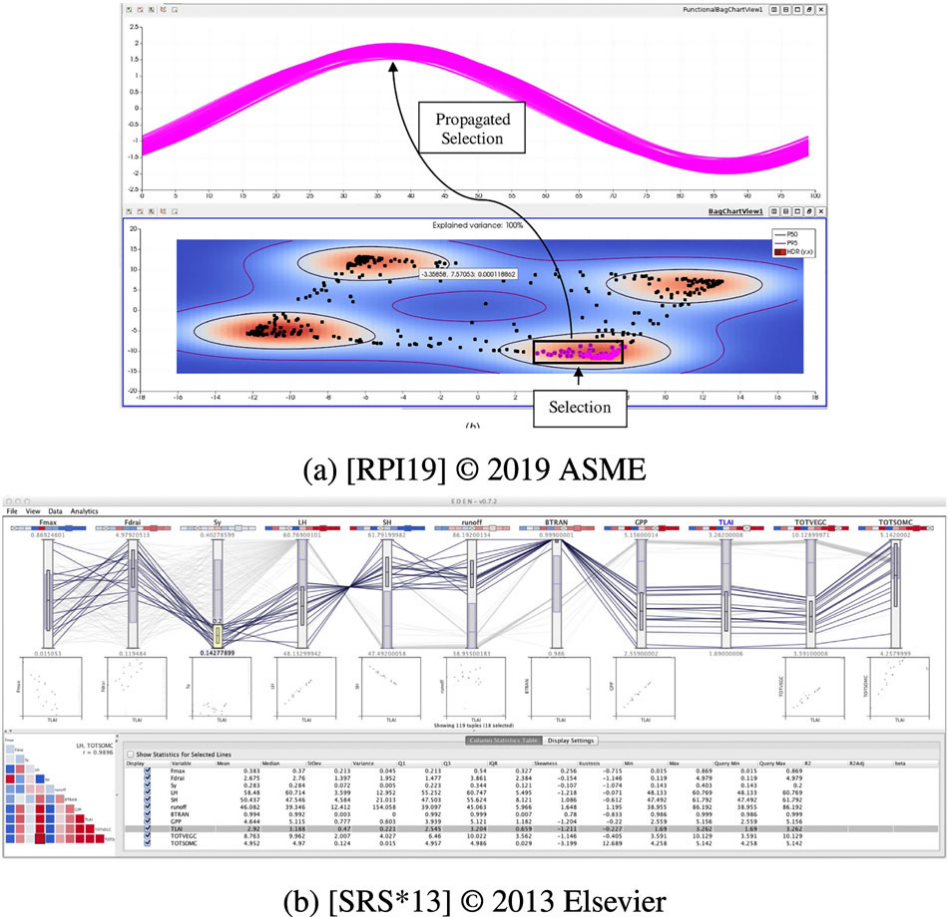
Examples for Focusing (Section 7.1). (a) Focus on individual data cases (time series) by selection. (b) Focus on multiple data cases by filtering.

This theme collects interactions where the analyst focuses on a subset of data cases through *selection*/brushing (item‐based) or *filtering* (attribute‐based) or on a region/interval of interest through *navigation* in time or space. In other words, they decide to either look at fewer data cases or less information about a single data case (or both). By selection, individual data cases are marked as interesting. When relevant abstract attribute ranges are defined, it is referred to as filtering or attribute‐based selection. Finally, space and time often need to be navigated independently of attribute values. See Figure 19 for examples. Overview+detail visualizations [CKB09] can be used to maintain the broader context of the current focal region. Focusing on subsets of data cases or time/space is, on the one hand, necessary because display resolution and size are limited. On the other hand, a typical parameter space analysis process requires Focusing interactions. Input/output visualizations (Section 6) display parameters and outputs while highlighting relations relevant to the required parameter space analysis task, for example, optimization or sensitivity analysis. To go from such *findings* to *insights* and *knowledge* [SSS*14], analysts have to, for example, inspect relevant data in more detail or find related data cases, which they achieve with interactions discussed in this section.

##### Select

Selection is often performed by clicking on a data case in a specific visualization, which could, for example, be a ranking [WKS*14, SOL*16] or a time‐varying vector field [SJJ*17].

Selecting multiple data cases can be achieved by grouping them first (Section 7.5) and then allowing selection on the group representatives [BM10, BHGK14, FMH16], or by classical multiple selection tools, like a lasso [WFR*10]. In systems with multiple linked views, this functionality is provided by brushing and linking (Figure 19a). The inverse operation to selection is available in some works, where the user can exclude data cases from the analysis [OBJ16, YDH*17, SWO*20].

##### Filter

Picking out individual data cases is cumbersome or infeasible when there are many. In such a case, a solution is to define a filter on their attributes. This approach is ubiquitous with systems that employ multiple linked views. An often‐used example [MGJ*10, MDG*13, MGS*14, MAJH17, CKS*17] of those is ComVis [MFGH08], which allows flexible brushing and linking in any view. Such systems allow analysts to filter in either parameter or output space and see the effect on the other. Parallel coordinates (Figure 19b) and related visualizations are especially common for this task [SRS*13, CLEK13, BHGK14, DFL*15, OKB*19, RPI19, AE20], possibly after feature derivation (Section 7.2), but so are histograms and scatterplots. In a multiple‐linked view system, InfoVis can be combined with spatial/temporal data. For example, Ribičić et al. [RWF*13] use them to present derived features (Section 7.2) from spatio‐temporal flood simulations. After the analyst selects data cases by brushing, related frames from multiple simulations are highlighted in a World Lines view [WFR*10]. Analysts are provided sculpturing‐inspired tools that allow them to filter 3D models based on spatial features in the DreamLens system [MGB*18]. For example, the “chisel” tool defines a line in 3D and excludes any mesh that intersects that line.

##### Navigate Time

With temporal data, it is natural that analysts focus on a subset of the time axis because temporal data may span a long interval or have high resolution. This task is often solved by zooming into a smaller contiguous interval [BHR*19]. When there is additionally a spatial dimension in the data, it may be possible to either look at a summary of all temporal data in space (and vice versa) or to inspect single time steps in more detail [BLLS17]. The latter can be simplified by segmenting the time series and showing representatives [BM10, BWMM15].

##### Navigate Space

We can look at the dimensionality of the part of interest to further categorize focusing in space beyond geometric view transformations such as pan/zoom or rotation. There are points, lines, surfaces, areas, and volumes. Points of interest occur, for example, in particle simulations [GT16, SJJ*17], where analysts may place seed points for particles and inspect their trajectories, but also in lighting design, where designers place glare probes in a room [WSL*20]. Schultz et al. [SK13] filter vertices of a 3D mesh by any existing or derived scalar value at a vertex by selecting thresholds in a density plot. Areas of interest, of course, naturally appear with two‐dimensional spatial data. For example, in image segmentation, Pretorius et al. [PZR15] allow to brush a subset of reference images so that analysts may focus on known problematic regions. Areas in 3D are surfaces and classified into usage types (e.g., work, leisure) in the context of lighting design [SOL*16, WSL*20] to verify legally prescribed light conditions. For Hazarika et al. [HLW*20], the space is a circle (an idealized yeast cell), and hence the interesting part is a line around it. Analysts may select a portion of that circle by brushing and querying for parameter settings that maximize/minimize the yeast simulation response there. Axis‐aligned cubes of interest are used by Amirkhanov et al. [AHRG10] to mark features in a 3D scan.

### 7.2 Derivation

**Figure 20:**
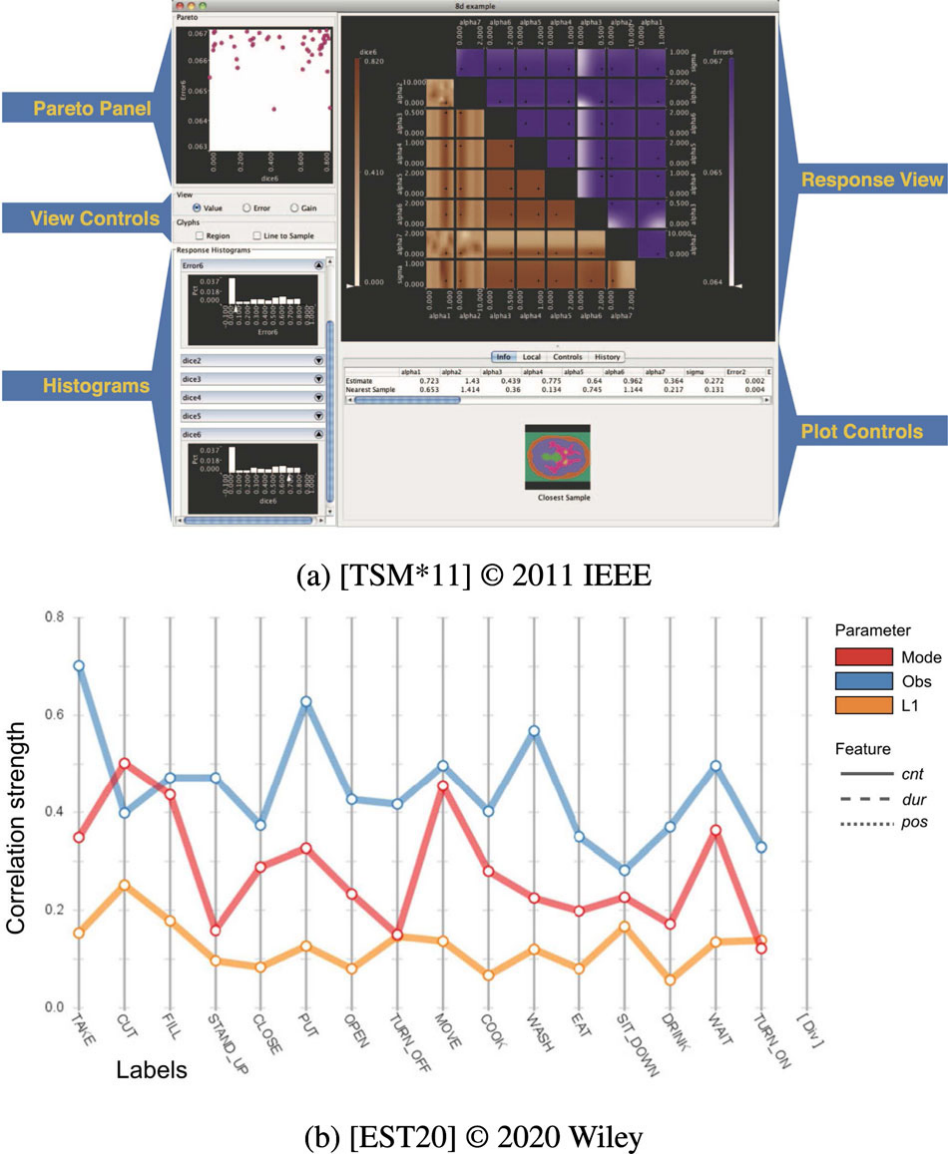
Examples for Derivation (Section 7.2). (a) Two measures of similarity between segmented image (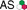 output, bottom) and reference segmentation in a HyperSlice visualization of an 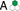 parameter (top right Response View). Dark areas mark high quality of outputs, hence supporting parameter *optimization*. (b) Parallel Coordinates Plot showing correlations (Y position) between a 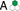 parameter (line) and the number of segments with a given label (axes), a derived feature from the 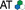 output of a time series segmentation model. It is visible that the Obs parameter influences the number of labelled segments most (*sensitivity*).

We refer to Derivation when new, simpler information is generated from a single data case. Usually, this data case is the output, and we call the result a feature. We classify information that does not pertain to a single element but a population thereof (e.g., central elements, distributions) as Aggregation (Section 7.3). Derived features are often scalars that quantify something of interest, such as how well an output matches a “ground truth” reference. Derived features may also preserve the spatial/temporal dimension. For example, when boundaries of homogeneous regions in an image are of interest, those might be found with an edge detection algorithm. See Figure 20 for examples.

Usually, features that quantify output characteristics are domain‐ and application‐specific, so it is not helpful to list them here. On the other hand, when comparing to a reference, several metrics can be used. These are distance or similarity metrics; the difference between the two is that the former fulfills the triangle inequality while the latter does not. The distinction may be necessary for algorithms working with relative distances between elements, as some, such as k‐means [Llo82] or HDBSCAN [CMS13], require the triangle inequality to hold and may be used only with distance metrics. Similarity metrics exist for different data types, such as multivariate data (Euclidean/Minkowski distance), text (edit distance), sets (Dice/Jaccard/Tversky index), matrices (norms), polygons (Hausdorff or Fréchet distance), or images (structural similarity index measure), to name some examples.

##### Scalars Quantifying Output Features

Derived features in this category quantify domain‐specific features in the output and produce one or more scalar values (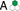). These features are various. From the visual appearance of 3D models (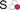 output) [MGB*18] to how far sandbags (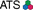 parameter) were swept by a flood (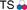 output) from their initial position [RWF*13]. Energy use can be derived from a building design (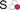 output) [AE20], and the amount or length of labels from a time series segmentation (Figure 20b, [EST20]). Well‐known summary statistics are also used, like minimum/maximum value of a time series [MGJ*10, MGS*14].

##### Scalars Quantifying Output Quality

The other group of scalars (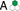) quantifies the output quality. If no inherent quality metric exists, for example, the number of intersecting triangles of a 3D mesh, outputs can be compared to a reference (“ground truth”). The latter can come, for example, from human experts (Figure 20a, [TSM*11]), from actual physical measurements, like the arrival time and speed on earth of a coronal mass ejection [BPM*15], or from government regulations, like lighting conditions in a work environment [WSL*20]. The former group of scalars depends on the application domain, and proper derivation functions have been identified for image segmentations [FMH16], porosity analysis in materials [WAG*16], or 3D meshes [BHGK14].

##### To Time+Abstract Data

When the aforementioned scalars are derived per time step of a parameter/output with temporal characteristics, one derives 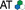 data. They fall into the same two categories, that is, they quantify either output quality or characteristics. Uncertainty in time was quantified by Biswas et al. [BLLS17] to show how a spatio‐temporal model is influenced by grid size (a model parameter) and by Bernard et al. [BHR*19] to highlight which parts of a multivariate time series change significantly by a preprocessing algorithm. Similarly, Röhlig et al. [RLK*15] and Luboschik et al. [LRE*12] show the fit to a reference over time. Many features that indicate spatial and group behavior in the context of chaotic movement patterns in biology were plotted over time in another work by Luboschik et al. [LRB*15]

##### To Space+Abstract Data

Derived features may also preserve the spatial dimension, producing 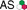 data. Malik et al. [MHG10] used edge detection to highlight differences between many 3D X‐ray computed tomography images. Sagristà et al. [SJS20] detect ridges in a finite‐time Lyapunov exponent field. Obermaier et al. [OBJ16] derive metrics about temporal and spatial trend characteristics.

### 7.3 Aggregation

**Figure 21:**
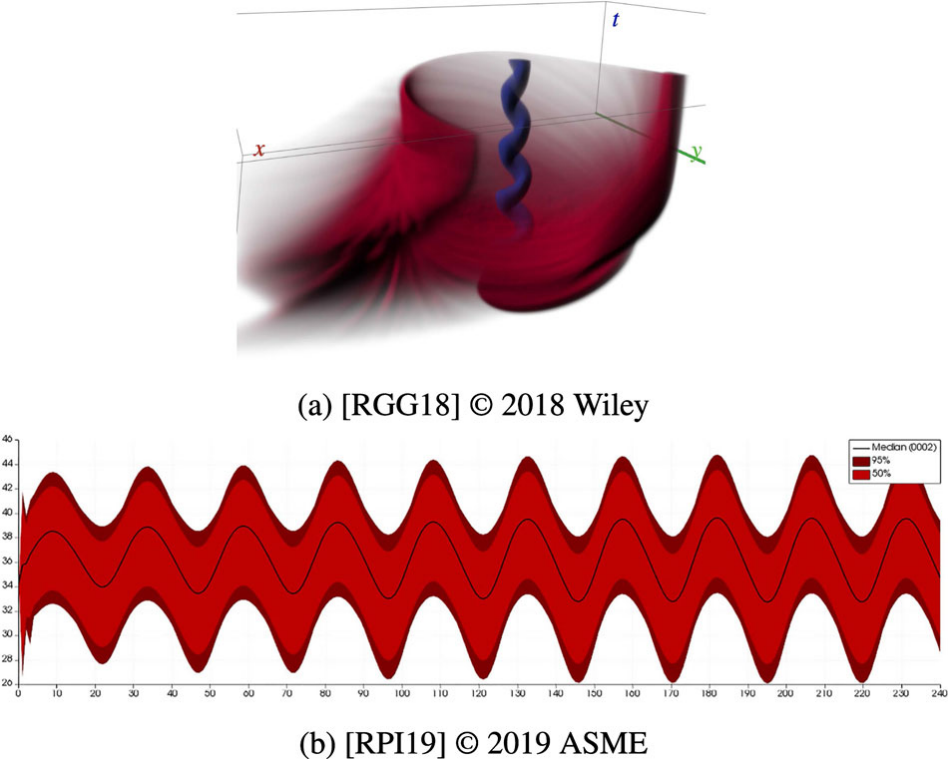
Examples for Aggregation (Section 7.3). (a) A density plot in a spacetime cube shows the distribution of particle trajectories (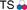 output) with identical initial location but varying velocity and size (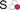, 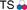, 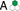 parameters). The blue spiral marks small particles of size 100μm, and the red blob around it particles of size 300μm, thus highlighting common behaviour within each particle size (*partitioning*). (b) Plot of median time series and quantiles shows most frequent temporal behavior of 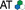 outputs.

Multiple data cases are aggregated in one way or another to reveal information related to statistical distributions, for example, central items, outliers, or frequency of items. Data characteristics of data cases are retained, that is, aggregating many time series yields a time series, and aggregating scalars yields a scalar. Classic examples for 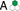 data are summary statistics, like mean or standard deviation, histograms, box plots. Distributions in time and/or space are also often of interest. Naturally, as this section is about summarizing spatial and temporal data, overlap with approaches used in ensemble visualization [WHLS19] is expected. Focusing on the common behaviour of multiple elements while preserving data characteristics sets this sub‐theme apart from Derivation (Section 7.2). We distinguish between characteristics of aggregated data. Examples are depicted in Figure 21.

##### Abstract Data

Abstract data often arises as part of feature derivation (Section 7.2). Matković et al. [MGJ*10] summarize many time series (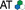) by showing a histogram of a 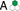 user‐defined feature (minimum, average, or maximum value). Sagristà et al. [SJS20] summarize a finite‐time Lyapunov exponent (FTLE) field by counting ridges (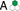 feature), which are then aggregated by summary statistics. Unger et al. [USKD12] use the average goodness‐of‐fit of a geoscientific simulation model to uncertain ground truth to validate the model.

##### Space

Landesberger et al. [vLBK*13] show a 2D distribution plot of 3D meshes (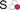 static input) so that the analyst may choose between a gaussian and non‐gaussian distribution (a parameter of the 3D segmentation algorithm). To summarize stochastic 3D packings of molecules (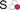), Schwarzl et al. [SAJ*19] use a density plot from an orthogonal direction.

##### Space+Abstract

Beham et al. [BHGK14], as well as Fröhler et al. [FMH16], aggregate multiple image segmentations (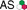 output) to a single visualization image by highlighting where segmentations disagree. Cibulski et al. [CKS*17] summarize a set of surfaces (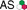 output) with 3D boxplots. Raidou et al. [RCM*16] show uncertain regions of tumour treatment by showing the variability of recommended radiation dosage from multiple parametrizations of a tumour control probability model (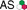 output). Malik et al. [MHG10] perform edge detection on scanned images, yielding a 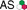 feature, then align histograms to the side of a scan that shows how many images have an edge in that row/column but not others.

##### Time+Abstract

Ribés et al. [RPI19] find quantile time series by density analysis in principal component space of many 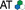 simulation outputs (Figure 21b). Bernard et al. [BDB*16] highlight the uncertain parts of multiple time series segmentations (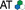) by showing the probability of class labels over time with line graphs.

##### Space+Time

Rojo et al. [RGG18] employ density volumes and isosurfaces to show the distribution of particle trajectories (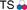 output) in time and space (Figure 21a). By separating the density volumes further using colour (cf. Section 6.3), the influence of the particle size (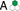 parameter) becomes visible (*partitioning*, *sensitivity* tasks). Sagristà et al. [SJJ*17] use phase‐space FTLE maps to show the variance of particle trajectories (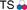 output) depending on the initial position or initial velocity. To analyze many flood simulations (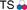 output), Ribičić et al. [RWF*13] propose an aggregation pipeline that involves extraction, grouping, aggregation, and embedding.

### 7.4 Sorting

**Figure 22:**
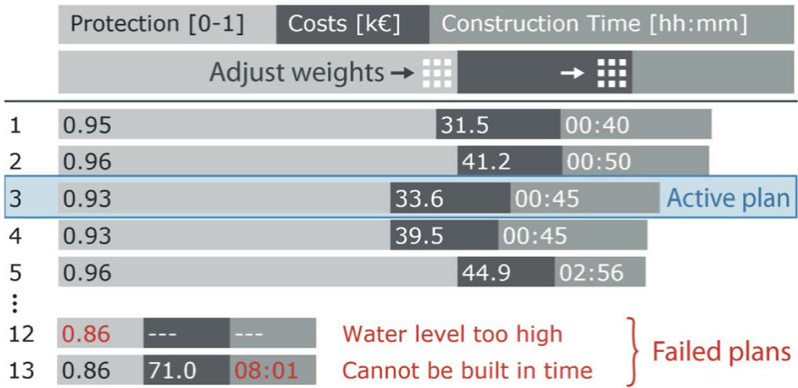
Example for Sorting theme (Section 7.4): Ranking of derived 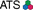 output features of a flood simulation in the form of a list. Only top‐ranked solutions (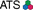 parameter settings) are relevant for the *optimization* task as they protect many buildings and may be constructed in time. [WKS*14] © 2014 Wiley

Another approach to reducing the amount of data cases is to rank them according to some logic. As position in space is the most accurate visual variable, sorting parameters/outputs allows organizing complex data quickly and aids understanding as the analyst only needs to inspect the top few results (Figure 22). When sorted data cases are presented as visual objects, for example, glyphs, complex patterns may become apparent (cf. Section 6.4). We distinguish sorting by scalars (one‐dimensional 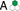 data) and complex (i.e., everything else) attributes.

##### By Scalar

Producing a 1D ordering of objects is known as seriation [Lii10]. The simplest case is a 1D seriation of a scalar, which we can sort. Arrangements along a single dimension include lists, rankings, and so on, but also spreadsheets sorted by one column. In the context of flood simulations, Waser et al. used this technique to sort parallel universes based on a derived 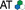 simulation state [WFR*10]. Approaches making use of design galleries also often allow sorting those based on a user‐defined criterion, like the value of a derived feature [DLC*15, MGB*18]. In interactive optimization, Liu et al. [LDT*21] recommend sorting obtained solutions by the objective function's value.

##### By Complex Attribute

Sorting by more complex data than scalars was also often used to organize data cases. For example, a user‐defined weighted sum of a multidimensional 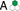 attribute produces a scalar again. Waser et al. ranked protection plans (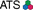 parameter) by a weighted sum of cost, protection, and construction time (Figure 22a, [WKS*14]). Sorger et al. [SOL*16] ranked lighted scenes (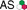 output) by how close lighting conditions are to legally prescribed values on surfaces of interest. With spreadsheet‐based visualizations, parameters and outputs (or derived features) are represented as columns and data cases as rows. Users may then sort all rows or subsets by one or more columns. As temporal/spatial data make up one column, different similarity metrics and sorting algorithms, for example, optimal leaf ordering [HHB08], can be used to obtain an ordering. Spreadsheet‐based approaches have been mostly used with 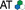 outputs and 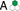 parameters [LRE*12, LRHS14, RLK*15, BBB*18, EST20], but also with derived features from spatial data [PZR15], or spatio‐temporal data [LRB*15].

### 7.5 Grouping

**Figure 23:**
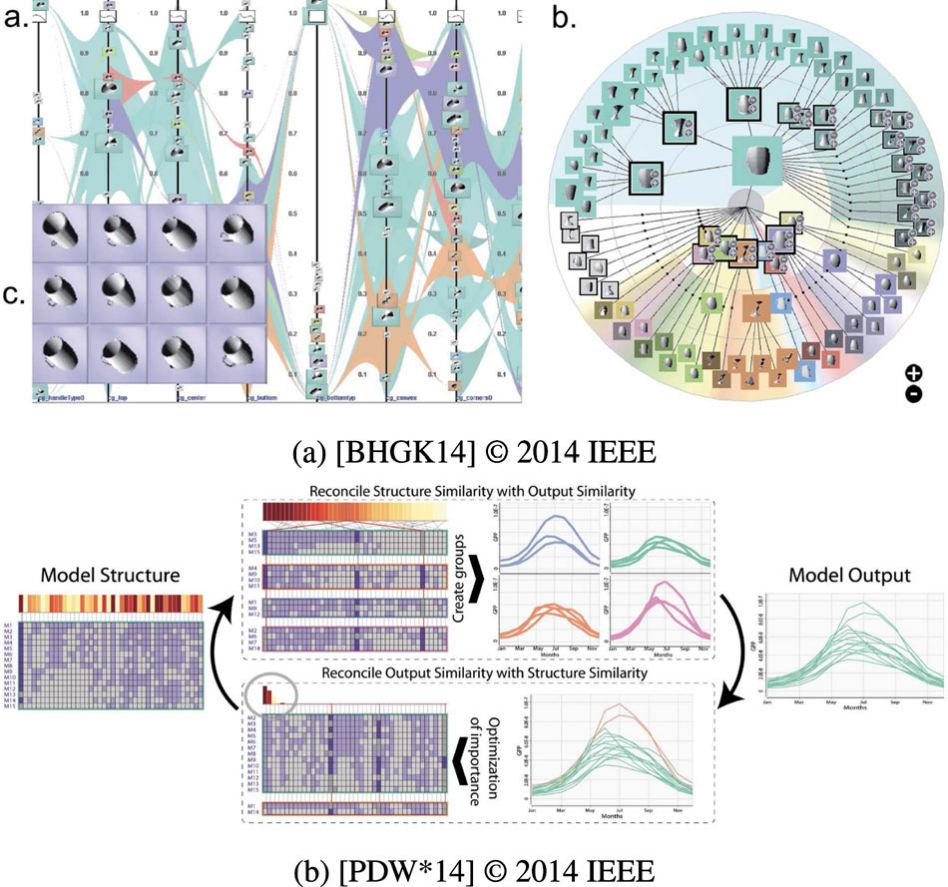
Examples for Grouping (Section 7.5). (a) Clustering (right) by 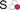 outputs was used for a 3D cup generator. Associated parameter settings for clusters are shown to the left in the Parallel Coordinates Plot, supporting *sensitivity* analysis. (b) Analysts may group time series (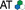 outputs) by 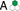 simulation parameter, thus carrying out a *partitioning* task.

Separating data cases into coherent groups is another way to organize a large body of data (Figure 23). This task can be achieved automatically through clustering algorithms if similarity information of data cases is available. It may also make sense to let the user decide on the particular groups, which are then formed based on the current analysis goal. For example, authors often used Grouping to *partition* the output space and, by visualizing parameter settings per cluster, showing their *sensitivity*. We distinguish further by which characteristics data cases are grouped.

##### By Abstract Data

A hierarchy of 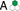 parameter settings is used in Paramorama [PBCR11], allowing analysts to quickly step through relevant subspaces (subtrees). The parameter space of Poco et al. is a binary vector (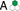). Hence, analysts may group the 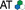 outputs by whether a Boolean parameter is set or not (Figure 23b, [PDW*14]). Different groups of data cases appear automatically in the work by Bao et al. [BYMW13] as the dimensionality of underlying parameter subspaces makes it necessary to present data cases separately. When 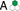 features are derived from outputs (cf. Section 7.2), grouping by abstract data may also involve outputs [OBJ16, BLLS17]. Notable is Schulz et al. [SWG*18] who, in parametric engineering design, achieved a partitioning of solutions in performance space (stress, mass, heat) where groups contain Pareto‐optimal designs.

##### By Space

Abuzuraiq and Erhan [AE20] use hierarchical clustering both on the 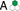 parameter space and on the resulting 3D shape (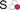) of the building in the context of generative architecture. Similarly, Beham et al. (Figure 23a, [BHGK14]) group 3D meshes (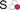 output of a generative model) and display emerging clusters in a 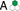 parameter space visualization (PCP). Fröhler et al. [FMH16] group image segmentations (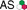 output) hierarchically and visualize their disagreement. Clusters may be selected (Section 7.1), which updates linked 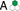 parameter visualizations. In parametric design, Woodbury et al. [WMCM17] allows analysts to group 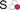 data cases (3D models) into collections, which may be automatically expanded by combining 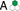 parameter settings.

##### By Space+Time

Ribičić et al. [RWF*13], in their proposed pipeline to visualize data from multiple 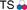 flood simulations, group data in a domain‐specific way and distinguish between objects (buildings), fields (water) and instances (sandbags). Information about group members is subsequently aggregated (Section 7.3) and visualized, for example, by Embedding (Section 6.3). Working in visual effects design, Bruckner et al. [BM10] group 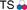 outputs into coherent temporal segments based on frame similarity. The segments are then depicted in a timeline.

## 8 (Surrogate) Model Tuning

In some VPSE systems found in the literature, it is possible to interact with the (actual or surrogate) model itself. In some applications, this is necessary because building a suitable model is part of the parameter *optimization* task. An example is pipelines, a common concept in image [WAG*16] or time series processing [BHR*19]. The analyst needs to find appropriate parameter settings and choose the required steps (e.g., outlier removal or smoothing), their order, and which algorithm to use. We can distinguish the operation performed on the model: Editing and inspecting. The former alters the model, while the latter collects and presents its internal information. Papers are summarized in Table 5.

**Table 5:** Combined overview of Provenance and (Surrogate) Model Tuning themes. Rows are ordered by number of papers. A filled dot • means the theme applies, an empty dot ○ means it does not. 72 papers without either theme were omitted.

#		Provenance	(Surrogate) Model Tuning
19	[TSM*11, WDR11, BAF*13, SK13, Mar13, BAF*14, DFL*15, ZSN*15, OAH15, FMH16, BBGM17, YDH*17, KGC*17, MGB*18, WSL*20, SWO*20, DTSO20, KSG20, LDT*21]	•	○
8	[MGJ*10, vLBK*13, MGS*14, BWMM15, WAG*16, MAJH17, BHR*19, HLW*20]	○	•
2	[BRG*12, DLC*15]	•	•
29	∑	21	11

### 8.1 Editing

Editing refers to the previous example of building a pipeline or a surrogate model inside the system as part of the exploration process. The latter was done in two works by Matković et al. [MGS*14, MAJH17], where the analyst defines a regression model on a data subset. This model was then further used to estimate and sample a 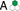 parameter subspace [MGS*14]. As for pipelines, we found examples for image processing [vLBK*13, WAG*16] and time series processing [BRG*12, BHR*19]. Matković et al. [MGJ*10], in the context of an electronic unit injector simulation, allow the analyst to build a schematic model of the individual involved components. Bryan et al. [BWMM15] support analysts in defining a suitable emulator for a complex simulation with 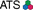 output. Finally, Dang et al. [DLC*15] assist an analyst in defining a probability density function for a shape grammar, in which, after user interactions, they automatically update probabilities of individual rules and the set of rules themselves.

### 8.2 Inspecting

Inspecting, on the other hand, exposes the internals of the surrogate model to the user. While inspecting model internals can be required to build a proper surrogate, it was sometimes also used on its own. Matković et al. [MAJH17] show regression coefficients of a user‐developed surrogate model to quantify relationships between 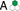 parameters and 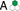 features derived from 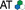 output. Hazarika et al. [HLW*20] visualize weight matrices of the neural network surrogate model to validate that it learned domain‐aligned logic.

## 9 Provenance

The term “provenance” [XOW*20] in the visualization literature roughly refers to tracking either how data was generated/modified or how the user interacted with the system. The former is referred to as *data provenance* [BKWC01], while the latter is known as *analytic provenance* [NCE*11]. Papers in our survey used exclusively analytic provenance (Table 5). Capturing and using user interactions is useful, for example, for an analyst to recall the analysis process. Within VPSE, we can more specifically distinguish between the following approaches:

- Analysts mark single data cases that appear in a dedicated list (bookmarks) [TSM*11, DFL*15, OAH15, FMH16, SWO*20, DTSO20];
- systems that capture every intermediate result [BAF*13, BAF*14, ZSN*15, BBGM17, WSL*20, LDT*21];
- load/save functionality to recover past work [WDR11, BRG*12];
- giving names to individual data cases [YDH*17].

Thus, this theme refers mainly to accessing relevant data cases later. While other reasons for collecting and using provenance data can be found in the context of VPSE, they were rather few. In only one instance [SK13] was interaction history not used for bookmarking but for replicating useful parameter settings on other datasets. Data provenance was not used at all, which is maybe not surprising given that investigated data often come from simulations and their heritage is thus well known.

## 10 Relations to Other Taxonomies

Models in papers of our survey do not *exclusively* encompass spatial/temporal inputs/outputs, but also include 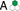 data (compare Section 2). Hence, it is not surprising that they used many strategies suggested for 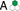 data [BM13, Mun14]. Even more so because spatial/temporal data are often “reinterpreted” as 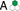 data. For example, the phase of a particle (location and velocity) can be seen as a 4‐tuple of [X, Y, angle, speed], and a time series as a long vector. Further, feature derivation (Section 7.2) is another common strategy to convert temporal/spatial data to 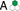 data. For example, 3D building models (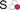) to performance metrics (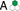), vector fields (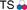) to the number of ridges (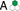), or time series (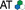) to the maximum value (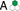). Consequently, strategies to interact with and visualize abstract data become even more enticing during visualization design in our context. For these reasons, we will discuss the relations of our themes to Brehmer and Munzner's multi‐level typology for visualization tasks [BM13]. Additionally, the InfoVis pipeline by Card et al. [CMS99] is a widely accepted model of how raw data is transformed to a visualization image (Figure 1, top right). As such, discussing the connections between pipeline steps and our themes will clarify the latter and aid understanding.

### Multi‐Level Typology for Visualization Tasks

Our theme **Finding Parameter Settings** may be best compared to an *import* task, as it leads to new data cases available in the system. **Input/Output Visualization** contains ways to *encode* input and output variables in visualizations, but the task is mostly carried out by the visualization designer, not the user. Themes in **Data Case Organization** largely overlap with the *how* part in Brehmer and Munzner's typology. Focusing encompasses *select*, *filter*, and *navigate* tasks, and Derivation is a *derive* task. Sorting is different from *arrange* in the typology because Brehmer and Munzner see it as changing relative positions of entire views, for example, with multiple coordinated views, whereas we refer to sorting data cases. Thus it is more akin to an *encode* task, or *arrange* in Munzner's book [Mun14]. Our Grouping does not seem to have a counterpart in Brehmer and Munzner's typology. A *selection* creates only two groups (selected and not selected), and so does *filter*. The group membership could be seen as a *derived* variable in their taxonomy, but we do not make the same requirement about persistence. Also, our Aggregation sub‐theme differs slightly from an *aggregate* task. Brehmer and Munzner define it as “methods that change the granularity of visualization elements,” that is, a view transformation in the InfoVis pipeline [CMS99], while in our case, it is about data transformations. The two remaining themes **(Surrogate) Model Tuning** and **Provenance** are again best described by *import* in Brehmer and Munzner's typology. Tuning the (surrogate) model necessitates recomputation for existing inputs and thus adds new data cases to the system. The same is true when previously saved data cases are loaded, whereas bookmarks are a *record* task.

### InfoVis Pipeline

The majority of our themes may be seen as *data transformations* in that model: **Finding Parameter Settings** is about obtaining new parameter/output pairs, hence it adds rows to the underlying data table. **Data Case Organization** aims to simplify the data under investigation in various ways. As such, it enhances data tables by new columns (e.g., cluster labels or sorting order). Filtering and navigation may be understood as limiting data table rows to relevant subsets, either by exclusion (*data transformation*) or by selecting single data cases for detailed inspection (*view transformation*). Changing the model itself (**(Surrogate) Model Tuning**) either leads to new rows in data tables, representing the updated relationship between inputs and outputs, or updating these relations in place. Hence, it may also be considered a *data transformation*, as can **Provenance**, which adds columns (e.g., “bookmarked?”) to data tables. Finally, only **Input/Output Visualization** targets *visual abstractions* and *visual mappings* as it describes how inputs and outputs may be visualized.

## 11 Open Challenges and Future Work

We identified areas for future work in the field through a systematic search and analysis of the literature on VPSE user interfaces, where either parameters or output reference space or time. Although both the literature search and the analysis are subjective to various extents, and the set of reviewed papers is not necessarily complete, we are confident to have collected a representative sample of relevant papers that allows drawing conclusions.

We contextualize our directions on future work for the field with those by Sedlmair et al. [SHB*14], who looked at VPSE from a more system‐centric perspective. Their identified research gaps pertained to data acquisition, data analysis, and cognition. Data acquisition is about the ability to obtain interesting parameter/output pairs within the VPSE system. The data analysis gap refers to “opening the black box” specifically for the derivation/prediction steps in their data flow model. The cognition gap is about how to facilitate the search for and navigation between 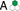 parameters. Other mentioned future work topics were scalability, guidance, provenance, collaboration, and evaluation.

Regarding guidance, Ceneda et al. [CGM*17, CGM*18, CGM19] defined it as a computer‐assisted process that resolves a knowledge gap of the analyst in an interactive VA session. It received lots of attention in recent years [CAS*18, SJB*20]. The knowledge gap in VPSE usually relates to parameters (*data* domain), that is, which settings cause the most certain/optimal/sensitive/outlying outputs, so it should not be surprising that many of our themes are associated with certain characteristics of guidance and vice versa. A few examples: *Orienting* guidance often involves visual clues. Hence it can be found in our Input/Output Visualization and Data Case Organization themes. The Manual/Constrained sub‐theme is related to *directing* guidance when the system presents options to choose from and *prescribing* guidance when it automatically adapts solutions or prohibits selection outside of certain parameter subspaces. The domain of the knowledge gap is mostly the *data* (parameters/output pairs). Some works [KSSI17, KGS19, KSG20], which break the parameter selection problem down to simpler sub‐tasks, can also be seen to provide a solution in the *tasks* domain. The guidance input is usually the *data*, but examples exist for others, for example, *domain knowledge* [WKS*14] or *user knowledge* [PBCR11]. Our increased understanding of guidance since the survey by Sedlmair et al. [SHB*14] shows us that it has been there since the beginning [JKM00, TSM*11], albeit sometimes in subtle ways. Thus, the question for the future is less about how to provide guidance for VPSE, as we have provided many examples in this survey. Rather, it is about fine‐tuning the guidance process and making it more flexible, for example, combining multiple guidance inputs, timing guidance correctly [CAGM21], switching between guidance degrees [PCE*22] and means to show the answer, and so on.

However, in our view, other topics (scalability, provenance, collaboration, evaluation) are for the most part still current, even though our perspective is different, as we focus on the user interface. We will list our topics for future work in VPSE first and afterwards relate them to those by Sedlmair et al. [SHB*14].

### Parameter Space Tasks in Time and Space

We collected 101 papers supporting various VPSE tasks for models where *either or both* parameters and outputs have a temporal/spatial reference. A complete table of papers, including referenced space/time characteristics, can be found in the supplemental material. Slicing this dataset in different ways, we find chunks smaller than others and thus indicative of gaps in the literature. Table 6 shows a contingency table of parameter space analysis tasks and data characteristics of the parameters. The row margins show that most papers discuss 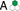 (63/101) or 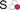 parameters (27). At the same time, we found only a few papers for the remaining space, time, and abstract combinations. Naturally, some parameter space tasks remain unsupported for these combinations (9 cells highlighted in red). For 20 other combinations, there are only a few examples in the literature. We highlight the relevant cells of Table 6 with three or fewer examples in light orange. Hence, future work should investigate the tasks uncertainty analysis, partitioning, outliers, and fitting for 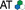, 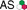, and 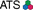 parameters. More generally, VPSE systems for other than 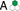 or 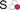 parameters seem rare enough to warrant future explorations.

**Table 6:** Contingency table of parameter space tasks [SHB*14] (columns) and parameter type (rows), where A = abstract, S = space, and T = time. Red colour highlights task/parameter combinations that were not tackled by any paper in our survey. Light orange highlights combinations tackled by 1–3 papers. Note that a VA system may support multiple tasks (cf. Table 1) and a model may require multiple parameters.

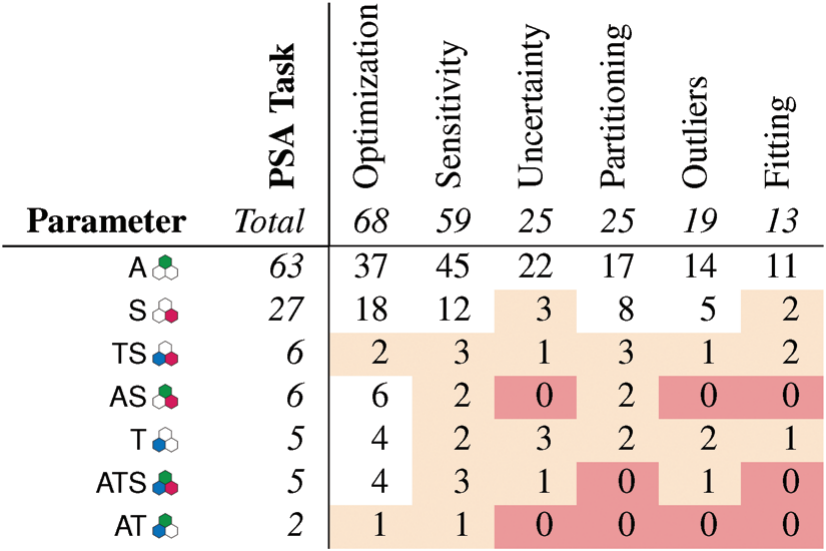

### Data Volume

The larger collections of data we saw were about a few thousand parameter settings and relatively small associated data, for example, 3D models of a monitor stand. Our survey gives relatively few answers how to enable VPSE for data‐intensive models, where the output of a single run is on the order of gigabytes of data. He et al. [HWG*20] suggest a possible approach, in which the surrogate model skips the output and learns the visualization image directly. Producing partial results during model execution (Progressive Visual Analytics [ASSS18]) might be another viable strategy to build interactive visualizations for data‐intensive models.

### Data Variety

Most of the models in our survey take one or a few parameters and produce a single output. We did not see data structures such as graphs, sets, hierarchies, or even multiple outputs a lot. This may be due to simplifications introduced by visualization designers or an actual property of many models. In any case it is an open question how to enable VPSE for such inputs/outputs.

### Data Quality, Data Provenance, and Uncertainty

Many models take complex input parameters, such as time series. These input parameters may need to fulfill some properties, for example, the time series being free of holes (no missing values). It may also be the case that the original input did not have these properties and was preprocessed somehow to this end. Few works consider the uncertainty introduced by such preprocessing steps, or uncertainty that may have existed in the input from the beginning. This is an important future research direction towards reliable and trustworthy insights with VPSE.

### Analytic Provenance

The Provenance theme in our survey is about quickly accessing individual data cases, as that is the part of provenance‐related interactions that was mostly exposed to users. Xu et al. [XOW*20] reviewed provenance in visualization and identified several ends to which provenance data was used. We saw in our survey approaches for *model steering* [Mar13, KSSI17, KGS19, KSG20] and *replication* [SK13], but others, like *adaptive systems* or *understanding user* are less explored. In which ways analytic provenance can be leveraged for VPSE is, therefore, an interesting research direction for the future.

### Composite Visualizations

We classified visualizations that show model inputs and outputs in Section 6. From Table 3 it can be seen that the majority used Juxtaposition, which speaks to the flexibility of the approach. Some composition approaches were used seldomly or rarely, for example, Integration, Nesting, Overloading. This suggests that the design space of composite visualizations in VPSE is not fully explored yet and future work in this direction might uncover useful visualization idioms.

### Data Organization Approaches

It can be seen from Table 4 that Sorting and Grouping are the least popular sub‐themes in that category. That is somewhat surprising because these two approaches are part of the basic organization activities we do in everyday life. For example, when organizing a bookshelf, we often group by book owner and sort by author. While related tasks are different—quick retrieval (bookshelf) versus pattern perception (parameter analysis)—VPSE by flexible grouping and sorting of data cases should be explored more, given how intuitive the two actions are.

### Advanced Interaction Design

Woodbury and Mohiuddin [WMCM17, MW20] suggest that designers prefer to pursue multiple design alternatives in parallel and to quickly explore alternatives. We only found one system besides theirs that really allowed that [ZSN*15], where users edited graphs of drawing operations for a 2D pattern. How VPSE users can work simultaneously on other complex models and how to quickly come up with suitable alternatives of complex parameters is another promising research direction for the future. In a biological simulation context [HLW*20] it was suggested that this interaction paradigm may be useful not only for designers. A so far not taken direction could be grammars, which encode rules how to construct complex objects from simpler parts [ARB07, GLXJ14, ZXKL*26]. Additionally, most surveyed works employed the established WIMP paradigm (windows, icons, menus, pointers). Exploring VPSE with alternative paradigms, like in virtual reality [BBGS16], or input devices, such as tablets [KGC*17], encompasses another direction for future research.

### Collaborative Aspects

Most surveyed papers were intended for a single user working on one machine. Collaborative aspects were seldomly considered in the proposed systems. A part of Visdom [KWS*14] is dedicated to justifying decisions to avoid flood damages, for example, where to put barriers, so that officials may explain those to the public. How people can work together in a VPSE setting, is still mostly untouched territory.

### Opening the Black Box

Many papers in our survey saw their model as a black box and focused more on parameter/output relations instead of how the internals work. The many successful applications show that this approach works in general. It is especially advantageous, for example, when intermediate steps inside the model are not important or not well understood by analysts. In other cases, it may lead to better outcomes or deeper insights into how the model works. Future work should determine when and if the additional effort of the “opening” process (e.g., in terms of visualization design) is warranted. A few papers we surveyed considered a pipeline of processing steps, which could be viewed as opening up a model. Aside from that, VPSE designers may draw inspiration on how to open black box models from a large body of research about using VA to interpret machine learning models [CMJK20].

### Model Comparison

Most works investigate a single model. It is, however, not difficult to imagine that alternative models exist, for example, different segmentation pipelines [WAG*16], models with different assumptions [RBV*14], or different formulations of the same physical reality [HG18]. In our survey we found only few works that focus on the specific task of model comparison, for example, finding respective parameter subspaces that lead to comparable results. More research in this regard could help domain experts choose models based on other considerations than exactness of the output.

### Supporting Larger Data Processing Pipelines

Most of the models in systems we surveyed deal with a single step of a more extensive data processing pipeline. Even, for example, time series preprocessing, which is in itself a pipeline, is only at the beginning of a more holistic task. The larger pipeline also consists of several interdependent steps. Every step incurs choices regarding parameter settings or algorithms, influencing subsequent steps. Systems we found focused either on single pipeline steps and ignored the bigger picture or focused on the whole constructed pipeline and glossed over details. We believe the spectrum between the two extremes is worth exploring more.

### Evaluation Practices

Ultimately, we are all interested in what part of our visualization designs worked and what did not, which is why we evaluate our designs. VPSE fits mainly in the “Visual Data Analysis and Reasoning” scenario by Lam et al. [LBI*12]. Proposed evaluation practices include case studies, interviews, or controlled experiments. All of these involve human participants. However, half of the surveyed papers where we could infer that information reported no human participants (median 0.5, mean 4.39, standard deviation 7.83). This number is to be taken with a grain of salt, as our survey includes papers from various journals and conferences. Interactive visualizations for VPSE were not always the main contribution of the paper. Nevertheless, it suggests a certain imbalance between how VPSE systems *should* be evaluated and how it is done in practice. Future work should put more emphasis on appropriate evaluation practices of suggested designs and approaches to strengthen the body of knowledge of our community.

It is apparent that, although our survey took a different focus on VPSE, many topics from 2014 are still current [SHB*14]. In particular, our challenges of *data volume/variety* are close to *scalability*, *provenance* is in both lists, as are *collaboration* and *evaluation*. The *data analysis gap* called for opening the black box of the derivation and prediction step in their data flow model, and is closely related to our own *black box* challenge. However, there are differences, too. In our survey, we did not perceive the *data acquisition gap* as a pressing problem, because 71/101 VPSE systems allowed users to obtain new parameters within them. That is not to say that the gap is not an issue anymore, creative ways are needed to *scale* VPSE to models that are expensive in terms of processing power or storage. We saw some progress towards the *cognition gap*, for example, breaking the parameter selection problem down to more, but simpler tasks, seems like a promising direction [KSSI17, KGS19, KSG20]. As a consequence of our different survey focus, we were able to identify additional topics for future work that were so far not mentioned, such as the need for *advanced interaction design*.

## 12 Conclusion

In this survey we focused on user interfaces for VPSE and on computational models with a temporal or spatial component. We did not restrict ourselves to a particular type of model or data, other than the space and time criterion, and, therefore, included a diverse set of papers. We identified several themes in how proposed systems work (Figure 1), which can be seen as a common workflow for VPSE systems. We presented those themes in more detail. New exciting directions for future research were identified (Section 11), while many from a previous survey with different focus [SHB*14] are still current. In this survey, we identified various approaches and ideas to VPSE together in order to see the whole picture. We expect that our work can help the theoretical analysis of VPSE and facilitate the development of novel techniques.

## Supporting information

Supporting Information
