# Supplementary material for: Visual Parameter Space Exploration in Time and Space
Source: Comput Graph Forum. 2023 Apr 3;42(6):e14785. doi: 10.1111/cgf.14785 (PMC10947302; doi:10.1111/cgf.14785)
Supplement: Supplementary file 1 — Supporting Information [file CGF-42-0-s001.zip › codebook_combined.pdf]

Final Codebook from Thematic Analysis

This document contains our final codebook that we developed as part of the thematic analysis process. It differs from the themes presented in our paper in a few ways, as we optimized for presentation later on.

- The *comparison* theme is not part of the paper. It tracks which part of the model (static/dynamic inputs, outputs, ground truth) is compared to each other to achieve the parameter space analysis task. We felt that this is not particularly helpful information and thus did not include it in the final themes.
- We also tracked how many parameter settings are handled by the system at a given point in time, and whether new parameters overwrite existing ones. We touch upon this briefly in the paper. In contrast to the paper, in the codebook this is part of the *finding parameter settings* theme.
- As we mention in the paper, we excluded the *data pipeline* theme due to low perceived scientific value.

paper	extract	theme	sub-theme	comment
koyama2014	smart suggestions	finding parameter settings	manual/constrained, automatic/supervised	
	sliders and a single view	comparison	input + output	
	scented widgets	comparison	inputs + outputs	
	user interaction with sliders	finding parameter settings	manual/unconstrained	
	sliders show fitness function along one dimension			
	visualizations highlight good part of param space and how quality changes along 1 dimension	psa task	optimization, sensitivity	
	output vis = image, input vis = visopt sliders	visualization	sequential superposition	
	visopt slider = parameter slider + output quality	visualization	align	
luboschik2014	sort rows based on any column	data case organization	sorting	
	change temporal or parameter scale	data case organization	focus	navigate
	main spreadsheet/matrix style vis	comparison	inputs + outputs	
		psa task	fitting, outlier, sensitity, uncertainty, optimization	per sedlmair 2014
	spreadsheet vis	visualization	align	
beham2014	radial cluster view	data case organization	group	
	color links cluster view + pcp, small regression lines	comparison	inputs + outputs	
	cluster view shows different possible models	comparison	outputs	
	highlight cluster in PCP, show 3d shapes in detail view	data case organization	focus	select
	different icons for cups	data case organization	aggregate	
	parameter description	finding parameter settings	automatic/unsupervised	
	geometric properties	data case organization	derive features	
	task 1 (find similar shapes and their params)	psa task	partitioning	
	task 2 (find errors and implausible shapes)	psa task	outlier	
	task 3 (sensitivity of parameters)	psa task	sensitivity	
	color (output simil.) in PCP	visualization	embed	
	little icons in pcp	visualization	overload	
poco2014	side by side views	comparison	inputs + outputs	
	split into groups	data case organization	group	
	goal is in the end to figure out which (combination of) flags in input space lead to what sort of outputs	psa task	sensitivity	
	matrix view, line chart view	visualization	juxtapose	
sorger2016	3d view	comparison	output + ground truth	
	select a few settings for more detailed comparison	data case organization	focus	navigate+select
	group and label surfaces ("work area")	data case organization	focus	filter space
	view where it shows how much deviation from target	comparison	outputs + ground truth	
	ranking + icicle plot and target values	data case organization	sorting	
	target values on texels	data case organization	derive features	
	main goal is to compare multiple outputs and decide for one based on many tradeoffs	psa task	optimization	
ruppert2014	analyze view with tons of bar charts	comparison	single output	
	enter policy	finding parameter settings	1 edit manual/unconstrained	
	explore view	comparison	inputs + outputs	
	fig 5 align output vis in grid of input dimensions	visualization	align	
doraiswamy2015	analyst can decide how real buildings are approximated, set weights for view score	finding parameter settings	automatic/supervised N add	
	export polygons	data pipeline		
	filter designs by individual window view scores	data case organization	focus	filter
	building / window view mode	comparison	single output	
	design cart	provenance		
		comparison	outputs	
	pcp and gallery	comparison	inputs + outputs	
	use case 1: figure out when the view starts improving	psa task	sensitivity	
	in that system, view quality is the main metric to judge a building	psa task	optimization	
	output heatmap on input model	visualization	embed	
	pcp of outputs + gallery of 3d models	visualization	juxtaposition	
matkovic2014	analyst can decide which parameter to vary how	finding parameter settings	N add automatic/unsupervised	
	features are extensively used to make the regression work	data case organization	derive features	
	histograms	data case organization	aggregate	
	multiple linked views with brushing	comparison	inputs + outputs	
	brushing	data case organization	focus	select
	select subset of outputs, define regression model	model building		
	use regression model to suggest optimal values	finding parameter settings		
	table 1, r3	psa task	optimization	
	table 1, a1	psa task	sensitivity	
	table 1, r1	psa task	uncertainty	they say "accuracy" but it's really about prediction uncertainty
	build regression model	psa task	fitting	
	parameter histograms with optimum value as polyline			
	comvis	visualization	juxtaposition	
weissenbock2016	define segmentation pipeline	model building		
	define parameter ranges	finding parameter settings	N add automatic/	
	choose to compare models or compare datasets	comparison	between-model comparison	
	pre-filter and also later find model/dataset combination	data case organization	focus	filter
	porosity values	data case organization	derive features	
	paramter range slider	comparison	inputs + outputs	
	reference porosity	comparison	outputs + ground truth	
	boxplots, histograms	data case organization	aggregate	
	define ROI in slices	data case organization	focus	navigate
	2d slice overlays	comparison	outputs	
	they say so themselves in related work	psa task	optimization, partitioning, sensitivity	
	colored dots by output quality in scatterplot of inputs	visualization	embed	
	popup preview	visualization	overload	
	this orange thing in figure 7	visualization	integrate	
yumer2015	interaction with sliders	finding parameter settings	manual/unconstrained 1 edit	
		comparison	inputs + outputs, input + output	
	attribute map	comparison	outputs	
	sliders, attribute map can highlight variance in cars	psa task	sensitivity	
	sliders can be locked	psa task	optimization	
	attribute map shows cars arranged in 2d by how 'sporty' they are	psa task	partitioning	since you can look at the high/low areas and browse what sort of cars are there
	a lot is based on "fiddling"	visualization	sequential superposition	esp. fig 3a
	attribute map = 2d projected heatmap + 3d model with "arrow" to position on heatmap	visualization	integrate	
frohler2016	define parameter ranges	finding parameter settings	N add automatic/unsupervised	
	tree view from hierarchical clustering	data case organization	group	
	object count	data case organization	derive features	
	select group of items by clicking cluster in tree	data case organization	focus	select
	histograms, example images from cluster	data case organization	aggregate	
	cluster member agreement view	comparison	outputs	
	large view of currently selected image	comparison	single output	
	scatterplot, brushing in histograms	comparison	inputs + outputs	
	favorite bar	provenance		
	lens	?	?	is it very important though
	self-provided assessment	psa task	partitioning, sensitivity	
	input/output scatterplots	visualization	embed	
	parameters next to output views	visualization	juxtapose	
	lens of original image in segmentation view	visualization	overload	because it's offset a bit
obermaier2016	trend graph	data case organization	group	partition by time interval first, then by similarity
	deactivate members in parameter space view	data case organization	focus	filter
	scalar quantities	data case organization	derive features	
	parameter space view + the others	comparison	inputs + outputs	
	trend graph gives overview of temporal behavior	psa task	partitioning	
	parameter view shows where in input space the output occurs	psa task	sensitivity	
	parameter view next to other views	visualization	juxtapose	
luboschik2015	features extraction central to everything here	data case organization	derive features	
	reorder rows acc. to columns	data case organization	sorting	
	select 1 data case	data case organization	focus	select+navigate
	detail view	comparison	single output	
	matrix view	comparison	inputs + outputs	
	line chart	comparison	outputs	
		psa task		same vis idiom as luboschik2014, so same tasks
	spreadsheet idiom	visualization	align	
desai2019	manual parameter input supported by perception relationship curves	finding parameter settings	manual/unconstrained	
	pick from example gallery	finding parameter settings	manual/constrained, automatic/supervised	
	automatic slider	finding parameter settings	manual/constrained	unsure how this should score on the machine dimension
	scented widgets	comparison	inputs + outputs	
	side by side sliders and robot	comparison	input + output	
	lock slider values	psa task	optimize	
	little line charts on top of paramter values	psa task	sensitivity	
	separate parameter view + large single of output	visualization	sequential superposition	
	parameter view has regression line to output semantic attribute	visualization	superimpose	
walch2020	tree view with treemaps and screenshots and so	provenance		
	taget light values	data case organization	derive features	
	floor group, desk group	data case organization	focus	filter space
	select one setting for detailed view	data case organization	focus	select
	direct manipulation of lights etc	finding parameter settings	1 edit manual/unconstrained	i think a series of manual changes are captured as 1 in the tree, hence there's no undo, hence it's with replacement
	automatic suggestions	finding parameter settings	automatic/supervised, manual/constrained	
	single 3d simulation view	comparison	output	
	treemaps and screenshots in provenance view	comparison	inputs + outputs	

	self-provided assessment	psa task	optimization, partitioning	
	lights sources (parameters) are shown within the lighted scene (output)	visualization	superimpose	
berseth2021	define how much floor plan elements can change, define objective, ROI	finding parameter settings	1 add automatic/supervised	
		psa task	optimization	
	only output visible	visualization	-	
liu2018b	presentation mode	comparison	output	
	comparison mode	comparison	outputs	
	select plans to present or compare	data case organization	focus	select
	planning mode, set target values	finding parameter settings	N add automatic/supervised	
	manual editing, lock and reoptimize	finding parameter settings	manual/constrained	
	compare mode: show radiation dose (output) and seeds (param)	visualization	superimpose	
hazarika2020	sensitivity vis, max/min brushes, inspect NN layer	comparison	inputs + outputs	
	quick view toggled to show difference	comparison	outputs	
	the brushes allow to filter based on features in space	data case organization	focus	select/filter depending on your opinion
	max/min brush	finding parameter settings	automatic/supervised	
	manual input	finding parameter settings	1 edit manual/unconstrained	
	there are dendrograms	data case organization	group	the 400 points in the circle are clustered based on predicted value and uncertainty
	self-provided assessment	psa task	optimization, sensitivity, uncertainty	
	parameter sensitivity view with the 35 concentric circlular heatmaps (1 per param)	visualization	superimpose	
	separate view for parameters	visualization	juxtapose	although i'm not 100% sure that the parameter view is actually simultaneously visible with the activation view
liu2021b	reco 1	comparison	input	
	reco 2	finding parameter settings	automatic/supervised	
	reco 3	finding parameter settings	manual/unconstrained	
	reco 4	data case organization	sorting	
	reco 5	finding parameter settings	manual/constrained	
	reco 6	comparison	inputs	
	pick solutions for detailed (comparison) view	data case organization	focus	select
	reco 9	provenance		
		psa task	optimization	
	street map view shows parameters (# electricians, customers to be served, ordering constraints) and output (routes)	visualization	superimpose	
bernard2018	the visualization	comparison	inputs + outputs	
	sort rows	data case organization	sorting	
	uncertainty vis	comparison	outputs	
	uncertainty vis shows where output is stable	psa task	uncertainty	
	spreadsheet like vis	psa task	partitioning, sensitivity	
	spreadsheet like vis	visualization	align	
schulz2017	sliders and a single view	comparison	input + output, inputs + outputs	
		finding parameter settings	1 edit manual/unconstrained	
	goal is to find models that eg minimize stress	psa task	optimization	
	fiddling with sliders	psa task	sensitivity	
	fiddling with sliders	visualization	sequential superposition	
	heatmap on 3d model	visualization	embed	
umetani2014	manual input	finding parameter settings	1 edit manual/unconstrained	
	automatic optimization	finding parameter settings	manual/constrained, automatic/supervised	because they construct a cost function and find critical points of that function by numerical integration, so there's some objective that's optimized
	preview flight trajectories	comparison	input + output	
	edit view	comparison	input	
	by moving/changing the wings you should see a change in the trajectories	psa task	sensitivity	
	automatic optimization	psa task	optimization	
	see flight distance change while wing model is edited	visualization	sequential superposition	
cibulski2017	histograms, 3d boxplots, slices, heatmaps...	data case organization	aggregate	
	scalars can be aggregates (mean) or features (min of curve)	data case organization	derive features	
	multiple views	comparison	outputs	
		data case organization	focus	select
		comparison	inputs + outputs	
	task t1	psa task	partitioning	"get an overview"
	task t3	psa task	sensitivity	
	comvis system	visualization	juxtapose	
eichner2020	the three novel views	comparison	inputs + outputs	
	features of a segmentation	data case organization	derive features	
	in matrix view: highlight affected parameter subrange	data case organization	focus	select
	in matrix view: primary and secondary sorting	data case organization	sorting	
		psa task	sensitivity	
	tscv view	visualization	nested	
	spreadsheet view	visualization	align	
biswas2017		comparison	inputs + outputs	sensitivity dependent on time and space
		comparison	outputs + ground truth	accuracy
	change colormaps	view manipulation		
	select single day	data case organization	focus	select+navigate
	clustering	data case organization	group	
	RMSE	data case organization	derive features	
	error histograms	data case organization	aggregate	
	sensitivity analysis and prediction accuracy important topics	psa task	sensitivity, uncertainty	
	MDS view highlights outliers quite qell	psa task	outlier	
	no composite vis	visualization	-	
steiner2017	manual editing of floor plans	finding parameter settings	1 edit manual/unconstrained	
	repair bad structure	finding parameter settings	automatic/supervised	
	edit the thing	comparison	input	
	no composite vis	visualization	-	the FEM simulation renders are not part of the interactive system
gunther2016a	glyph vis	comparison	inputs + outputs	
	trajectories	comparison	outputs	
		finding parameter settings	automatic/unsupervised	
		finding parameter settings	N add manual/unconstrained	
	manually seed particles	data case organization	focus	navigate+select
	colored circle glyphs give an overview where particles land	psa task	partitioning	
	from the color-coding you can see areas that are not nearby the large ones	psa task	outlier	
	the coding also shows whether position, direction or velocity are the dominant factor	psa task	sensitivity	
	glyphs in grid show particle position and velocity and direction (parameter) plus where it ends up (output)	visualization	align	
	optionally trajectories can be superimposed	visualization	superimpose	
matkovic2017	brushing and selecting	data case organization	focus	select
	domain-specific features are used in discussion section	data case organization	derive features	
	build regression model	model building		
	build suitable regression model	psa task	uncertainty, fitting	accurate surrogate model = certain output
	look at factors of regression model	psa task	sensitivity	
	comvis	visualization	juxtapose	
schwarzl2019	select what parameters are exposed and how to sample	finding parameter settings	N add automatic/unsupervised	
	histograms, this flattened 3d view	data case organization	aggregate	
	brushing and selecting	data case organization	focus	select
	derived metrics	data case organization	derive features	
	export parameters	data pipeline		
	focus is on finding erroneous situations	psa task	outlier	
	parameters and outputs are next to each other in columns	visualization	align	
zaman2015	parallel edits, selective merging	finding parameter settings	manual/unconstrained	
	zoom/pan	data case organization	focus	navigate
	undo, restore from history	provenance		
	sampling of user-chosen parameter	finding parameter settings	automatic/unsupervised	
	duplicate setting	finding parameter settings	manual/unconstrained	
	optimization, sort of, because we look for subjective best setting	psa task	optimization	
	parameter view next to output	visualization	juxtaposition	
ribes2019	linked views	data case organization	focus	filter
	median curve	data case organization	aggregate	
	find common behavior	psa task	partitioning	
	find sensitive paraemters in PCP	psa task	sensitivity	
	1 view for time series, one pcp	visualization	juxtaposition	
ribicic2012	sketch uncertain breach location or water source	finding parameter settings	manual/unconstrained, automatic/unsupervised	
	uncertain breach location = uncertain parameter value	psa task	uncertainty	
	visualizing eg what range of water heights can be reached	psa task	sensitivity	
	everything is in the 3d scene, e.g., the amount of water coming from the ground simulation. many things have the 1:1 space mapping, like said water or also breach location etc.	visualization	superimpose	
	but there's also that arrow that indicates flow or whatever	visualization	overload	
chaudhuri2013	edit one 3d model, but only exchange parts and that also guided	finding parameter settings	1 edit manual/constrained	
	radial menu shows possible alternative parts	comparison	inputs + outputs	
	current 3d model is visible in detail view	comparison	1 output	
	radial menu says what metric is influenced by a part	psa task	sensitivity	
	design task	psa task	optimization	
	inputs (body parts) in radial menu over outputvis	visualization	overload	
unger2012	one parameter setting is analyzed in detail	comparison	output + reference	
	but several can be compared with aggregated goodness of fit	comparison	inputs + outputs	
	average fit	data case organization	aggregate	
	select samples	data case organization	focus	filter space
	goodness of fit vs model vs data view	comparison	outputs + ground truth	
		psa task		per sedlmair 2014

	model fit (Fig 7)	visualization	explicit encoding	
	colored scatterplots, cube etc (position = param, color = fitness = output)	visualization	embed	
konev2014	use domain specific rules to search solution space	finding parameter settings	automatic/supervised	
	decision cluster view	comparison	inputs	
	justification view	comparison	input + output	
	decision cluster view (fig 9b)	data case organization	sorting	arranged by temporal order and decision
	we look for barrier configurations that protect selected buildings	psa task	optimization	
	screenshots of simulation show water (out) and barriers (param)	visualization	superimpose	
waser2010	rubber band in vis mode	data case organization	focus	select
	order tracks based on simulation state	data case organization	sorting	
	branching, set parameter without branching	finding parameter settings	manual/unconstrained	
	vis mode	comparison	outputs	
	find barrier placements that protect buildings	psa task	optimization, sensitivity	per sedlmair 2014
	fig 3 track icon: simulation screenshot within WL track	visualization	nesting	
	fig 7: map color or height of WL track to output measure	visualization	embed	
malik2010	the nested hex view, the edge explorer	comparison	inputs + outputs	
	edge explorer histogram	data case organization	aggregate	
	edge detection	data case organization	derive features	
	nested hex thing	data case organization	sorting	
	move histogram lines, select zoom-in	data case organization	focus	navigate
	evaluation task 1-2	psa task	optimization	
	evaluation task 3	psa task	sensitivity	
	evaluation task 4-5	psa task	outlier	
	tile the spatial parameter (image) space, show outputs in different parts	visualization	superimpose	per gleicher 2011
	fig 7 b, c: parameter (label) and corr. feature in output are overlaid to current vis	visualization	overload	
bruckner2010	sampling relevant subspace			not done inside the tool
	select cluster item	data case organization	focus	select
	cluster similar segments	data case organization	group	
	cluster timeline, sequence view	comparison	outputs	
	parameter view	comparison	inputs + outputs	
	animation view	comparison	output	
		psa task	partitioning, optimization	from sedlmair 2014 classification
	parameter view is separate from rest	visualization	juxtapose	
kerr2010	the two slider options	finding parameter settings	manual/unconstrained	
		comparison	input + output	
		comparison	inputs + outputs	
	image navigation	finding parameter settings	manual/constrained	
		comparison	outputs	
	gallery view shows impact of 2 parameters	psa task	sensitivity	
	optimization, sort of, because we look for subjective best setting	psa task	optimization	
	image navigation layout: output images are arranged in a grid corresponding to two input parameters	visualization	align	
	other sliders: slider + output vis + fiddling	visualization	sequential superposition	
matkovic2010a	define the unit injector	model building		
	define which values are sampled	finding parameter settings	automatic/unsupervised N add	
	min/max	data case organization	derive features	
	brushing	data case organization	focus	select
	histograms	data case organization	aggregate	
		psa task		per sedlmair 2014
	comvis	visualization	juxtapose	
bernard2019	define pipeline	model building		
	effect of pipeline step on dimension	comparison	input + output	
	zoom/pan in time	data case organization	focus	navigate
	what does a varying parameter to single/all dimension	comparison	outputs	
	uncertainty quantification	data case organization	derive features	
	main goal is analysis of uncertainty introduced by preprocessing	psa task	uncertainty	
	superposed/aligned outputs are colored by parameter (fig 1)	visualization	embed	
	they are also shown superimposed (fig 1)	visualization	superimpose	
	and finally there's often a curve aligned underneath that shows the difference between original and processed (fig 1)	visualization	explicit encoding	
	alignment as per above	visualization	align	
he2020	sliders	finding parameter settings	manual/unconstrained	
		comparison	input + output	
	...with interaction	comparison	inputs + outputs	
	interactive sliders allow sensitivity analysis, and they compute some stuff too	psa task	sensitivity	
	mainly reconciliation happens with slider interaction	visualization	sequential superposition	
matejka2018	2d and 3d views, model stacking	comparison	outputs	
	filter outputs (by input or derived feature)	data case organization	focus	filter
		data case organization	derive features	
	attribute examples	comparison	outputs	data-org/partition used to do it
	multi attribute grid	comparison	inputs + derived outputs	glorified scatterplot
	design viewer = sorted gallery	data case organization	sorting	
	filter model stack	data case organization	focus	filter space
	ranking by example	data case organization	sorting	
	example task t1	psa task	optimization	
	example task t3	psa task	outlier	
	get representative examples for a given derived measure	psa task	partitoining	
	scatterplot can be configured with input param and derived measures	psa task	sensitivity	
	freely configure scatterplot	visualization	embed	
	attribute bars + output gallery	visualization	juxtaposition	
umetani2012	create, modify and join planks	finding parameter settings	manual/unconstrained	
	repair a broken setting	finding parameter settings	manual/constrained	
	suggestions	finding parameter settings	automatic/supervised	
	goal is to build a shelf that doesn't crumble	psa task	optimization	
	label shows if shelf is ok and why if not	visualization	overload	
swearngin2020	user sets high level constraints	finding parameter settings	automatic/supervised	
	delete designs	data case organization	focus	filter
	save/fav designs	provenance		
	import/export	data pipeline		
		comparison	outputs	
	system outputs diverse configurations that satisfy user-provided constraints	psa task	partitioning	
	parameters (spatial constraints) are juxtaposed to gallery	visualization	juxtaposition	
orban2019	virtual data instances	finding parameter settings	manual/unconstrained	
	move shit around in dr view	finding parameter settings	manual/constrained	
		comparison	inputs + outputs	
	parallel coordinates thing, nearest neighbours	data case organization	focus	filter
	example: "for each output curve there are multiple possible inputs"	psa task	sensitivity	
	2d DR views (in and out) next to each other	visualization	juxtaposition	
holbein2018	parameter ranges just exist? but i think they are sampled somehow	finding parameter settings	N, auto/	
	check influence of param on particle trajectory	psa task	sensitivity	
	compare parameter spaces of two models	psa task	-	there's no equivalent in sedlmair's taxonomy because they focus on single models
	spacetime cube vis with input param colored, cube vis with parameters of both models and measure of agreement in color	visualization	embed	
khan2019	save models	data pipeline		
	select by example using generative design approach	finding parameter settings	manual/constrained	
	performance metrics of hulls	data case organization	derive features	
	subjective optimization	psa task	optimization	
	no composite vis	visualization	-	
afzal2011	insert decision points	finding parameter settings	N, add, manual/unconstrained	
	cumulative lives saved/lost	data case organization	derive features	
	select 1 plan to show spatiotemporal behavior in detail	data case organization	focus	select
	decision view	comparison	outputs + inputs	
	detail view	comparison	outputs	
		psa task	optimization	per sedlmair 2014
	fig 7: input (decision) colored in chart of lives saved (derived from output)	visualization	embed	
	fig 7 thumbnails of simulation output	visualization	overload	
pretorius2011	users set up sampling	finding parameter settings	automatic/unsupervised	
	hierarchical ordering of params, reorder hierarchy levels, define cutoff, tagging	data case organization	group	
	select parts of hierarchy, filter by tag or param value	data case organization	focus	filter
	juxtaposed images	comparison	outputs	
	reference image	comparison	output + ground truth	
	hierarchy plus tags	comparison	inputs + outputs	
		psa task		per sedlmair 2014
	parameter hierarchy next to out output images (fig 4)	visualization	juxtapose	
	fig 4: manually decided output quality (green/pink color) shown in input parameter hierarchy	visualization	embed	
umetani2011	editing in 2d and 3d	finding parameter settings	edit, manual/unconstrained	
	change mannequin	data pipeline		
	view 2d template and 3d on mannequin	comparison	input + output	
	it's a design task, so sort of optimization on subjective criteria	psa task	optimization	
	optimizing done by altering 2d or 3d garment	visualization	sequential superposition	

torsney-weir2011	sampling points	finding parameter settings	N, automatic/unsupervised	
	dice coefficient and something else	data case organization	derive features	
	histograms	data case organization	aggregate	
	select a single point in the parameter space and inspect estimated values	data case organization	focus	navigate
	history and bookmarking	provenance		
	view response itself, gain and uncertainty	comparison	inputs + outputs	
	view 1 specific segmentation	comparison	output	
		psa task		per sedlmair 2014
	hyperslice vis of input + output measure via color	visualization	embed	
bao2013	load good examples	data pipeline		
	automatic search from the examples or other initial set	finding parameter settings	automatic/supervised	
	detail view	comparison	input	
	portal transitions	comparison	inputs	
	barycentric navigation in polygon, handle based thing	finding parameter settings	manual/constrained	
	the neighborhoods that the portals connect	data case organization	group	although that is more a technical necessity - each neighbourhood has a different dimensionality in the underlying parameter space
	find GOOD building	psa task	optimization	
	no composite vis	visualization	-	
coffey2013	one design is continually edited	finding parameter settings	1 edit	
	it can be changed at will (forward design)	finding parameter settings	manual/unconstrained	
	or so that it some output will be reached	finding parameter settings	manual/constrained	
	some derivations from a design are possible and used (wheel)	data case organization	derive features	
	1 large single	comparison	input+output	
	parameter wheel	comparison	inputs	
	output wheel	comparison	outputs	
	weights on wheels change which suggestions appear	data case organization	focus	filter
		psa task	optimization, fitting	from sedlmair 2014
	the bubbles are somehow doubly composite: first embed output on input, then superimpose several of such	visualization	superimpose, embed	
millward2013	load images, export analysis	data pipeline		
	set parameters of 1 cme event	finding parameter settings	1 edit manual/unconstrained	
	compare to different satellite images, leading edge vs time	comparison	output + ground truth	
	compare sliders to cone superimposed to satellite images	comparison	input + output	
	find best match to reference data	psa task	fitting, optimization	
	2d cone outline (output of the model) shown on top of satellite photos (input)	visualization	superimpose	
bogl2013	continual edits to one model	finding parameter settings	1 edit manual/unconstrained	
	but every change is tracked	provenance		
	... quantified with information criteria	comparison	inputs	
	residuals plot	comparison	outputs + ground truth	
	compare how current model is better/worse than previous	comparison	inputs + outputs	
	model diagnostics	comparison	input + output	
	information criterions	data case organization	derive features	
	box jenkins methodology contains objectives to follow	psa task	optimization	
	how and how much model deviates from original is relevant	psa task	uncertainty	
	residuals plot	visualization	explicit encoding	
	parameter view separated from rest	visualization	juxtapose	
ribicic2013	extract feature from frame (like water height)	data case organization	derive features	
	group values	data case organization	group	
	aggregate values within groups	data case organization	aggregate	
	brush and link	data case organization	focus	select
	embedding	comparison	outputs	
	parallel tracks, but here we assume they just exist	finding parameter settings	N	
	infovis and embedded views show a summary of future events	comparison	input + output	infovis views show frames with aggregated values, 3d views shows aggregated values embedded
	" When shown in a parallel- coordinates plot (Fig. 10g), two groups of outlier states stand out."	psa task	outlier	
	goal: stop flooding	psa task	optimization	
	"count operator, showing the area where water may spread, and how likely it is that an area will be flooded"	psa task	sensitivity	
	water (output) shown within scene (input)	visualization	superimpose	
	heatmaps on textures of inputs showing derived features of output (is building flooded?)	visualization	embed	
waser2014	analyst sets up scenario: region of interest, important buildings to protect, depots, how many trucks, etc	finding parameter settings	automatic/supervised	
	then analyst defines parameters and ranges	finding parameter settings	N add automatic/unsupervised	locations for barriers, barrier types, water height...
	cost weights influence ranking of solutions	data case organization	sorting	
	performance metrics for plans (cost time/money, protection)	data case organization	derive features	
	select 1 plan, show in big view	data case organization	focus	select
	ranking view	comparison	inputs + outputs	
	big single	comparison	input + output	
	goal: stop flooding	psa task	optimization	
	heatmaps of output on input texture	visualization	embed	
	water level (output) within scene (input)	visualization	superimpose	
bock2015	compare CME arrival time etc to reference, switch perf measure	data case organization	derive features	
	select 1 member	data case organization	focus	select
	timeline view, 3d view	comparison	output + ground truth	
	ensemble view	comparison	inputs + outputs + ground truth	
	find accurate ensemble members	psa task	uncertainty	
	compare model data with measured reference	psa task	fitting	
	ensemble view (fig 1) shows parameters of CME and fit to ground truth (measurement)	visualization	embed	
pretorius2015	sort table by params or measures	data case organization	sorting	
	filter data by param/measure ranges, select rows	data case organization	focus	select, filter
	images on the right	comparison	outputs + ground truth	
	table	comparison	inputs + outputs	derived outputs though
	derived outputs	data case organization	derive features	
	"it is straightforward to identify values for both that produce accurate nuclei detection (nucleus diameter takes its second value and threshold takes its first or second value)"	psa task	optimization	
	"Having established p2 as the parameter most clo- sely correlated with variation of the output measures"	psa task	sensitivity	
	table view (fig 1 left)	visualization	align	
	output images + input images (fig 1 right)	visualization	juxtapose	
schultz2013	continuously edit 1 spectral clustering	finding parameter settings	1 edit manual/unconstrained	
	filter points	data case organization	focus	filter
	rule sets	data pipeline		
		provenance		
	look at 1 segmentation	comparison	1 output	while it would be possible to e.g. change dimensionality of embedding and see how it changes result, the focus is on getting a good segmentation
	find paraemters that best segment the data	psa task	optimization	
	no composite vis	visualization	-	
rohlig2015	select and highlight rows	data case organization	focus	select
	sort columns	data case organization	sorting	
	switch encoding btween outcome and fit to reference	data case organization	derive features	
		comparison	inputs + outputs	
	accuracy (DR3)	psa task	uncertainty	
	relation parameter to label (DR2)	psa task	sensitivity	
	spreadsheet vis	visualization	align	
raidou2016	clinical researcher adds parameter sets	finding parameter settings	N add manual/unconstrained	
	brute force inverse search	finding parameter settings	N add automatic/supervised	
	probe TCP curve	data case organization	focus	select+navigate
	form groups of patients that have similar response patterns	data case organization	group	
	show variability of dose or TCP value	data case organization	aggregate	
	figure 5 line based param plot	comparison	inputs + outputs	uncertainty in output is what was interesting
	figure 8 heatmap of parameter combinations that are acceptable	comparison	inputs	
	figure 6 spatial view of dose	comparison	output	
	figure 6 spatial view of dose for multiple settings	comparison	outputs	
	tasks 1-3	psa task	uncertainty, sensitivity, partitioning	self-assessed apparently
	fig 5, fig 6	visualization	juxtapose	
odonovan2015	users edit 1 design continually	finding parameter settings	1 edit manual/unconstrained	
	but it can suggest stuff	finding parameter settings	automatic/supervised	
	and it also has a mode where suggestions are applied directly	finding parameter settings	manual/constrained	
	save intermediate layouts	provenance		
	large view	comparison	output	
	suggestions	comparison	outputs	
	goal: design	psa task	optimization	
	no composite vis	visualization	-	because each card as a whole is the output, no separate input vis
prevost2013	load base model, likely exportable too	data pipeline		
	define handles, minimum thickness	finding parameter settings	1 edit automatic/supervised	
	automatic optimization after user interaction	finding parameter settings	manual/constrained	
	see if 3d model is desirable	comparison	input	
	goal: make it stand lol	psa task	optimization	
	only shows models that stand, and "it stands" is not a property that needs visualizing, so no output vis	visualization	-	
merrell2011	make room, add furniture, move furniture et	finding parameter settings	1 edit manual/unconstrained	
	accept suggestions	finding parameter settings	manual/constrained	
		finding parameter settings	automatic/supervised	
	assess current design	comparison	output	
	compare with alternatives (in suggestions)	comparison	outputs	
	energy function based on design guidelines	psa task	optimization	
	focus is on the resulting scene, so no input vis	visualization	-	
bernard2016	users add new algorithms + params to vis	finding parameter settings	1 add manual/unconstrained	

	probability view shows multiple segmentations of a single class	data case organization	aggregate	
	select segments and results	data case organization	focus	select
	stacked colored segments	comparison	outputs	
	...show differences in models	comparison	between models	
	compare difference in segmentation results	psa task	sensitivity	
	multiple segmentations (outputs) ordered by algorithm group	visualization	align	
schulz2018	compute pareto neighborhoods	finding parameter settings	N add automatic/supervised	
	triangle vis shows areas of similar performance	comparison	outputs	
	hover over point in triangle	data case organization	focus	navigate
	see design on the right	comparison	1 input	
	high interactivity allows to compare between designs too	comparison	N inputs	
	effectively they obtain a partition of the performance space, no?	data case organization	group	
	video has also numbers of perf and design in the top left	comparison	1 input + output	
	we find pareto optimal designs	psa task	optimization	
	and that's how the perf space is partitioned	psa task	partitioning	
	browse pareto space of output measures by looking at various inputs in sequence	visualization	sequential superposition	
dayama2020	designer places elements, defines size and position	finding parameter settings	1 edit manual/unconstrained	
	accept suggestions	finding parameter settings	manual/constrained	
		finding parameter settings	automatic/supervised	
	save designs	provenance		
	you compare current design with alternative designs	comparison	outputs	
	you edit 1 design	comparison	outputs	
	design	psa task	optimization	
	input (canvas, workspace) shown next to gallery	visualization	juxtaposition	
marsault2013	main contribution is i think the interactive genetic algorithm framework	finding parameter settings	automatic/supervised	
	with that framework, generally newer generations replace older generations	finding parameter settings	edit	
	however, you can select some desirable examples and keep them around	finding parameter settings	add	
	you can steer that by selecting "nice" solutions	provenance		
	you can look at a 3d model in detail	comparison	input	
	you can look at different 3d models and also a small vis of their perf	comparison	inputs + outputs	
	i think only optimization can count here as it's using a GA that is nondeterministic	psa task	optimization	
	relative fitness underneath 3d model image	visualization	juxtapose	
sagrista2017	in: position+velocity, time. out: position+velocity			(position, velocity) = phase
	you can fix either initial position or velocity, then explore trajectories in the other space	finding parameter settings	1 edit manual/unconstrained	it's unconstrained because while one part is fixed, you can still set it to anything
	PS-FTLE-V/P maps help to identify ridges in final phase (and individual contributions of final position and velocity)	comparison	inputs + outputs	
	but you still have to fix one part of the phase, how to decide? stacked view	comparison	inputs + outputs	
	finally, the view i don't really get, multiplicity maps show you how many IVPs lead to that final position/velocity	comparison	inputs + outputs	
	add individual trajectories in the views	data case organization	focus	select+navigate
	the PS-TFLE-V/P maps partition input space based on variance in output space, sort of. or another view at it is that it's an aggregation (variance) of the output shown in input space.	data case organization	aggregate	just super complicated to do lol
	the maps show areas of different behavior, so you see all the possible behaviors of particles	psa task	partitioning	
	the areas also show how much you can change the initial position of a particle and get "qualitatively" equivalent behavior	psa task	sensitivity	
	fig 7a	visualization	align	
	trajectories, eg, fig 1 c,d	visualization	superimpose	
rojo2018	in: position+velocity+size, out: trajectory			
	change seed parameters	finding parameter settings	N add automatic/unsupervised	
	brush and link stuff, interactive seed	data case organization	focus	select, select+navigate
	iso surfaces, isosurface slabs, density views	data case organization	aggregate	
	one mainly sees trajectories or abstraction thereof, color-coded by particle size (an input)	comparison	inputs + outputs	
	how dependent is trajectory on particle size, position, velocity?	psa task	sensitivity	
	interactive seeding	visualization	superimpose	
	particles in same vis space at different heights (height = parameter, xy = plane, trajectory = output), color (parameter) in spacetime cube trajectory (output)	visualization	embed	
abuzuraiq2020	brushing and linking	data case organization	focus	select
	hierarchical clustering	data case organization	group	
		comparison	inputs + outputs	
	clsutering	psa task	partitioning	
	find best building design	psa task	optimization	
	scatterplot and pcp	psa task	sensitivity, outlier	
	pcp, scatterplot, gallery, dendrogram. can be reconfigured to show design (input) or performance (output) space.	visualization	juxtaposition	
bors2017	one edits the rastering until it's fine	finding parameter settings	1 edit manual/unconstrained	with direct manipulation interface
	quality metrics like how many empty rasters	data case organization	derive features	
	statistical summaries like how far away from original points	data case organization	aggregate	
	main view: original data plus the current rastering	comparison	input + output	
	history view	provenance		
	history view, quality view	comparison	inputs + outputs	
	original time series is the reference to which the output is compared to	comparison	output + ground truth	
	try to make the rastered time series not completely different from the original one	psa task	uncertainty	
	try to find the "best" rastering	psa task	optimization	
	superposed line charts of original and rastered series	visualization	superimpose	
schreck2012	users can influence how parameters are sampled, i suppose	finding parameter settings	1 add automatic/unsupervised	
	and also be set directly	finding parameter settings	1 edit manual/unconstrained	
	the small multiples view can be sorted acc to pattern similarity	data case organization	sorting	
	i understand you're mainly looking at the output, the classified points	comparison	outputs	
	check how "stable" interest points are with multiple parameter settings	psa task	sensitivity	
	fig 2 b	visualization	align	
	fig 1	visualization	superimpose	
bogl2014	users can set parameter of ts model	finding parameter settings	1 edit manual/unconstrained	
	comparison of reference (actual values) vs output (predicted)	comparison	output + ground truth	explicit encoding + superposition
	history of models	provenance		
	... quantified with information criteria	comparison	inputs	
	line graph with actual and predicted values	comparison	intput + output	
	find best prediction	psa task	optimization	
	prediction should be somewhat reliable	psa task	uncertinaty	
	compare actual and predicted values	psa task	fitting	
	parameter view separate	visualization	juxtapose	
	residual view = modeled - input	visualization	explicit encoding	
	output and input overlayed plus uncertainty	visualization	superimpose	
bernard2012a	build preprocessing pipeline	model building		
	pick time series from dataset to check pipeline impact	data case organization	focus	filter
	compare possible parametrizations of a routine on reference	comparison	input + outputs	
	lots of summary statistics	data case organization	derive features	
	save/load pipeline	provenance		
	manual input of routine params	finding parameter settings	1 edit manual/unconstrained	
	preview of param impact	finding parameter settings	automatic/unsupervised	
	find best preprocessing pipeilne	psa task	optimization	
	preprocessed time series should be truthful to the original	psa task	uncertainty	
	color-coded params in superposed view (fig 3)	visualization	embed	
	original ts and processed ts of single step are in same line chart (fig 3)	visualization	superimpose	
	fig 3: top has input in gray, bottom has various outputs	visualization	align	
yanez2017	define model parameters (how bad is the disease, how does it transmit etc)	model building		
	define countermeasures (school closures, vaccinations...)	finding parameter settings	1 add manual/unconstrained	
	label run	provenance		
	compare outcomes	comparison	output	
	technically i guess you could also change model parameters and compare outcome?	comparison	between models	
	parameter settings are labeled and outputs shown in same graphs	comparison	inputs + outputs	
	download simulations	data pipeline		unclear what is downloaded thought, but even if it's PDFs that's related to doing something else with the produced data
	available hospital beds, impact on workforce etc	data case organization	derive features	
	delete simulation	data case organization	focus	filter
	find best mitigation strategy	psa task	optimization	
	compare impact of mitigation measures	psa task	sensitivity	
	color-code param settings and show in output graphs (e.g. fig 7 b2, b4...)	visualization	embed	
hao2011	define 2 parameters of model	finding parameter settings	1 edit manual/unconstrained	
	look at input data	comparison	inputs	
	visual color accuracy indicator shows fit to historical data, find and compare time spans in input that were used to predict output, assess importance of input time spans for prediction	comparison	input + output	
	see predicted values	comparison	output	
	find best prediction	psa task	optimization	
	peaks are important for the application	psa task	outlier	
	uncertainty band shows reliability	psa task	uncertainty	
	accuracy indicator shows fit to past data	psa task	fitting	

	visual accurarcy indicator	visualization	align	
	interactivity by fiddling with params	visualization	sequential superposition	
raith2021	select eddies in either of the two views	data case organization	focus	select
	count ensemble members along parameters, time and space	data case organization	aggregate	
	compare sensitivity of eddie detection acc. to ensemble member, time and parameter setting, fig. 1 and disk glyph	comparison	outputs	
		psa task	uncertainty, sensitivity	
	layer on map, but glyphs don't share space with underlying layer and exact position is irrelevant as long as glyph is close to eddie	visualization	overload	
matkovic2010	analysis process: look at output data first and identify patterns, then refine and at some point look at control parameters	comparison	outputs	
		comparison	inputs + outputs	
	brush and link	data case organization	focus	select
	fig 4: derive numbers from space-time trajectories, eg max speed per segment	data case organization	derive features	
	fig 4: histogram	data case organization	aggregate	
	find fastest car configurations	psa task	optimization	
	figure out influence of car config on race	psa task	sensitivity	
	find uncommon laps/segments	psa task	outlier	
	see all behaviors	psa task	partitioning	
	comvis	visualization	juxtaposition	
matkovic2013	analysis process: look at output data first and identify patterns, then refine and at some point look at control parameters	comparison	outputs	
		comparison	inputs + outputs	
	filter data	data case organization	focus	filter
	brushing linking	data case organization	focus	select
	detail view for one run	comparison	output	
	find best car configuration for several objectives	psa task	optimization	
	comvis	visualization	juxtaposition	
gavrilescu2010	set parameters with sliders	finding parameter settings	1 edit manual/unconstrained	
	sliders show output variance in adjacent values	comparison	inputs + outputs	
	generally you see one slider setting and its output	comparison	input + output	
	vis highlights areas of parameter space where image changes a lot, ie, where small changes in param are associated to large changes in image	psa task	sensitivity	
	slider + output	visualization	sequential superposition	
	area or color used for output	visualization	embed	
steed2013	data selection panel involving space time dataset and variables	data case organization	focus	filter
	PCPs allows filtering	data case organization	focus	filter
	histograms underneath PCP	data case organization	aggregate	
	there is no reasonable distinction between parameters and result and everything is a 'variable' and you see all of them in the PCP, scatterplots and correlation matrix	comparison	inputs + outputs	
	scatterplots, pcp, correlation splom: we want to find correlations and dependencies between parameters and/or outputs	psa task	sensitivity	
	pcp contains parameter ("dataset") and output -> different variables in same vis	visualization	embed	
luboschik2012a	sort table by columns (parameter or accuracy)	data case organization	sorting	
	accuracy measures tell how close trajectory is to reference	data case organization	derive features	
		comparison	outputs + ground truth	
	single detail view shows average trajectory and reference	comparison	output + ground truth	
	aggregated accuracy measures shown next to paramter values	comparison	outputs + inputs	
	1000 runs per configuration	data case organization	aggregate	
	"experimenter is now able to choose a configuration that presents a suitable trade-off between execution time and accuracy requirements"	psa task	optimization	
	accuracy views, how close is the thing to the exact solution	psa task	sensitivity	
	aggregation over many trajectories along simulation time produces average trajectory	psa task	uncertainty	
	overview of all model outputs	psa task	partitioning, outlier	
	spreadsheet vis	visualization	align	
dang2015	modify parameters through a series of rating and automatic learning	finding parameter settings	1 edit manual/constrained	
		finding parameter settings	automatic/supervised	first it learns a feature space, then a similarity metric, then feature relevance, then a modified grammar
	select grammar examples to rate them	data case organization	focus	
	sorting by similarity	data case organization	sorting	
	rate examples	provenance		
	model (shape grammar) is continually adjusted	model building		
	i guess learning the feature space can be considered feature derivation?	data case organization	derive features	but is not under user control
	look at and rate one output	comparison	output	
	or several	comparison	outputs	
	goal: make suitable grammar	psa task	optimization	
	no composite i/o vis	visualization	-	
bryan2015	whole thing is about building emulators	model building		
	you compare the quality of emulators	comparison	between-model	
	and you assess single emulators with various measures, among of which is the run influence and information criterions etc	data case organization	derive features	
	the residuals basically are the diff between actual simulations and predicted simulations, spatial views	comparison	outputs + ground truth	
	show single prediction and just a few time points	data case organization	focus	select
	spatial analysis panel allows to compare predicted and actual runs	comparison	outputs	
		comparison	output + ground truth	
	user chooses from existing modeling approaches, can't set their parameters directly	finding parameter settings	1 edit manual/constrained	
	remove existing runs from model	data case organization	focus	filter
	goal: make suitable emulator of expensive simulation	psa task	optimization	
	to do so it might be necessary to prune overly influental runs	psa task	outlier	
	we are also interested in the prediction accuracy	psa task	uncertainty	
	and in the influence of individual parameters	psa task	sensitivity	
	fig 8: star coordinates + output	visualization	juxtaposition	
vonlandesberger2013a	goal: build pipeline for 3d segmentations	model building		
	preprocessing view (PCP, scatterplots)	finding parameter settings	1 manual/unconstrained edit automatic/unsupervised	
	preprocessing view	comparison	inputs + outputs	
	model selection = decide about distribution of traninig data	data case organization	aggregate	
	evaluation view	comparison	outputs + ground truth	
	mesh quality	data case organization	derive features	
	"The main task is the assessment of the impact of parameter choices on the output and the choice of the proper parameter setting"	psa task	optimization, sensitivity	
	fig 5	visualization	embed	
wu2011		comparison	input + output	input: small scale details in surface, material. out: large scale view
	continuous design of one solution	finding parameter settings	1 manual/unconstrained edit	
	load/save buttons	provenance		
	fiddling with sliders	psa task	sensitivity	
	goal: design good small scale structure	psa task	optimization	
	slider based definition of smalls scale structure	visualization	sequential superposition	
	figure 1	visualization	juxtapose	
amirkhanov2010	max/average ray penetration length, histograms	data case organization	aggregate	
	stability widget	comparison	inputs + outputs	
	3d view	comparison	input	assuming the 3d view shows the specimen properly rotated
	select region of interest	data case organization	focus	navigate
	color-coded triangle on 3d specimen	comparison	input + output	
	penetration length on a single projection	comparison	output	
	euler angles are automatically varied, probably, yes?	finding parameter settings	N add automatic/unsupervised	
		psa task		per sedlmair 2014
	fig 5	visualization	embed	
koyama2020	parameter setting through continuous selection from examples	finding parameter settings	1 edit manual/constrained	
	selection from gallery	comparison	outputs	
	previous selections HAVE to influence subsequent planes	provenance		
	output images arranged by (however rotated) plane in input space	visualization	align	
sagrista2020	possible parameters (T, t0) are precomputed	finding parameter settings	N	but i don't think it happens within the tool? they don't say
	then the FTLE field at a given (T,t0) coordinate is summarized	data case organization	derive features	somewhat similar to luboschik2015
	and then each derived feature is aggregated, too (max/avg/...)	data case organization	aggregate	
	region of interest definition by placing seed points	data case organization	focus	select+navigate
	one can look at one FTLE field with given params	comparison	1 output	
	the aggregation field shows inputs and aggregated derived output	comparison	N inputs/outputs	
	see all possible ridge behaviors	psa task	partitioning,outlier	
	see influence of t/t0 param on ridges	psa task	sensitivity	
	figure 1, left part with black/purple/yellow colormap	visualization	embed	
	seed points and trajectories, fig 1 middle part with blue colormap	visualization	superimpose	
geilinger2018	manual design mode	finding parameter settings	1 edit manual/unconstrained	
	semi-automatic design mode	finding parameter settings	1 edit manual/constrained	user edits shape of the robot, like length of feet (?), other parameters, like body angle or whatever, are automatically adjusted
	automatic optimization	finding parameter settings	automatic/supervised	change shape of robot automatically such that [something user-defined] holds
	one always reconciles robot shape and motion, i guess	comparison	input+output	
	we only work on output	visualization	-	
kazi2017	user specifies sketch freely, incl. design variables	finding parameter settings	manual/unconstrained	
	system samples design space	finding parameter settings	automatic/unsupervised	
	system finds optimal solution for each design variable setting	finding parameter settings	automatic/supervised	
	user browses then the obtained solution	finding parameter settings	N	
	user may then change design variables or other things	finding parameter settings	edit	
	UI shows derived stats such as volume etc	data case organization	derive features	

	user only ever sees one solution, so i guess it's automatic filtering to 1	data case organization	focus	select
		comparison	1 input/output	
	user can mark solutions to be inspected later	provenance		
	it's a design problem, so	psa task	optimization	
	one possible output of the simulation is overlaid on the sketch	visualization	superimpose	
mohiuddin2020	also a design problem	psa task	optimization	
	they use a PCP	visualization	embed	
	next to the PCP is a gallery	visualization	juxatpose	
	the PCP contains design variables and derived features from the output	data case organization	derive features	
	since it's a PCP you can technically do sensitivity analysis a bit	psa task	sensitivity	
	many inputs are specifeid	finding parameter settings	N	
	can be edited i guess	finding parameter settings	edit, add	
	only done by the user	finding parameter settings	manual/unconstrained	
		compare	N input/output	
woodbury2017	make/edit task	finding parameter settings	add, edit	
	select task	data case organization	group	
	expand task	finding parameter settings	automatic/unsupervised	
	many alternatives	finding parameter settings	N	
	user can produce any setting though	finding parameter settings	manual/unconstrained	
	pan/zoom on canvas	data case organization	focus	navigate
	derived features are shown as numbers underneath the design	data case organization	derive features	
	based on the screenshots i'd say comparison happens mostly on the images	compare	N outputs	
	but of course you can look at the numbers too	compare	N input/output	
	design task	psa task	optimization	
	fig 12: canvas has cards that show output image and a text table of inputs	visualization	juxtapose	
brunhart-lupo2016	brushing and selection	data case organization	focus	select
	request new outputs from selected input space	finding parameter settings	manual/unconstrained, automatic/unsupervised, N	
	"In order to explore the rich set of correlations in the simulation output,"	psa task	sensitivity	
	you only ever see one time step?	data case organization	focus	filter time
		compare	N input/output	
	inputs/outputs are in the same space with 3d polylines	visualization	embed	
johnson2019	"several important sub volumes", "specifying multiple critical times"	data case organization	focus	filter space, filter time
	row = view setting, column = simulation run, timestep = table	visualization	align	
	simulation output is flow particles, but they show lines	data case organization	aggregate	
	"highest stress on the lead occurs when the lead is at its stiffest", "the focus is on different lead lengths, which increase from left to right"	psa task	sensitivity	
	exploration, overview	psa task	partitioning	
wang2017	compare parameter correlations	psa task	sensitivity	
	compare accuracy of ensemble members	psa task	uncertainty	
	compare ensemble members to measurements	psa task	fitting	
	many simulation runs visible	finding parameter settings	N	
	use NPCP to subset data	data case organization	focus	filter
	NPCP and the rest are next to each other	visualization	juxtapose	in heatmap: rows = time, columns = member; PCP (input) next to heatmap (output) next to maps (output)
	accuracy quantification	data case organization	derive features	
lin2013	one setting continually edited	finding parameter settings	1 edit	
	inverse design: specify desired output, find parameter	finding parameter settings	manual/unconstrained, automatic/O	
	parameter shown in scene	visualization	superimpose	
		psa task	optimization	
raidou2014	cocoon integrates parameter ("choice"/"assumption") and derived features ("output parameter")	visualization	embed	
	next to cocoon there is a volume and slice view of the data	visualization	juxtapose	
	analysts want to find best choice	psa task	optimization	
	...know what impact choice has on outptu	psa task	sensitivity	
	...know which voxels behave in odd ways	psa task	outlier	
	analysts can highlight/brush	data case organization	focus	select
	the cocoon shows distribution of output per voxel and choice	data case organization	aggregate	
