# Supplementary material for: Visual Parameter Space Exploration in Time and Space
Source: Comput Graph Forum. 2023 Apr 3;42(6):e14785. doi: 10.1111/cgf.14785 (PMC10947302; doi:10.1111/cgf.14785)
Supplement: Supplementary file 1 — Supporting Information [file CGF-42-0-s001.zip › data_characteristics_wide.pdf]

paper	data-a	data-t	data-s	data-st	data-ta	data-sa	data-sta	param-a	param-t	param-s	param-st	param-ta	param-sa	param-sta	output-a	output-t	output-s	output-st	output-ta	output-sa	output-sta
101	*3*	*2*	*31*	*4*	*11*	*15*	*5*	*63*	*5*	*27*	*6*	*2*	*6*	*5*	*10*	*0*	*19*	*20*	*21*	*26*	*10*
sorger2016			x										x							x	
gunther2016a		x				x				x	x						x	x			
liu2021b				x				x										x			
amirkhanov2010			x							x										x	
bogl2013					x			x	x										x		
waser2010			x	x										x				x			
bruckner2010								x										x			
bernard2016					x			x											x		
schulz2017								x									x			x	
wang2017			x					x		x											x
dayama2020			x							x							x				
dang2015			x					x									x				
prevost2013										x					x						
beham2014								x									x				
bao2013										x					x						
abuzuraiq2020										x					x						
afzal2011	x		x									x									x
bernard2012a					x			x											x		
bernard2018					x			x											x		
bernard2019					x			x											x		
berseth2021			x							x										x	
biswas2017			x					x		x											x
bock2015										x	x							x			
bogl2014					x			x	x										x		
bors2017					x				x										x		
brunhart-lupo2016								x											x		
bryan2015	x						x	x													x
chaudhuri2013			x										x				x				
cibulski2017								x													x
coffey2013								x									x			x	
desai2019			x					x										x			
doraiswamy2015			x							x										x	
eichner2020					x			x											x		
frohler2016						x		x												x	
gavrilescu2010			x					x									x				
geilinger2018										x	x							x			
hao2011					x			x											x		
hazarika2020								x												x	
he2020								x									x				
holbein2018		x	x	x		x		x										x			
johnson2019			x					x		x								x			
kazi2017			x							x							x				
kerr2010			x					x												x	
khan2019								x									x				
konev2014			x	x										x				x			
koyama2014						x		x												x	

paper	data-a	data-t	data-s	data-st	data-ta	data-sa	data-sta	param-a	param-t	param-s	param-st	param-ta	param-sa	param-sta	output-a	output-t	output-s	output-st	output-ta	output-sa	output-sta
koyama2020						x		x												x	
lin2013			x										x							x	
liu2018b			x										x							x	
luboschik2015								x										x			
luboschik2012a								x											x		
luboschik2014								x											x		
malik2010			x					x												x	
marsault2013										x					x						
matejka2018								x									x				
matkovic2010								x										x			
matkovic2010a	x							x	x												x
matkovic2014								x							x				x		
matkovic2017								x											x		
matkovic2013								x											x		
merrell2011			x							x					x						
millward2013											x							x			
mohiuddin2020								x									x				
odonovan2015										x					x						
obermaier2016								x													x
orban2019								x											x		
poco2014								x											x		
pretorius2011						x		x												x	
pretorius2015						x		x												x	
raidou2016						x		x												x	
raidou2014						x		x												x	
raith2021							x	x												x	
ribes2019								x											x		
ribicic2013			x											x				x			
ribicic2012			x											x				x			
rohlig2015					x			x											x		
rojo2018							x	x		x	x							x			
ruppert2014								x											x		
sagrista2017						x				x	x							x			
sagrista2020							x		x	x								x			
schreck2012					x			x											x		
schultz2013						x		x												x	
schulz2018										x					x						
schwarzl2019								x									x				
steed2013												x									x
steiner2017			x							x										x	
swearngin2020			x							x							x				
torsney-weir2011						x		x												x	
umetani2012										x					x						
umetani2011			x							x							x				
umetani2014										x								x			
unger2012							x	x													x
vonlandesberger2						x		x												x	

paper	data-a	data-t	data-s	data-st	data-ta	data-sa	data-sta	param-a	param-t	param-s	param-st	param-ta	param-sa	param-sta	output-a	output-t	output-s	output-st	output-ta	output-sa	output-sta
walch2020			x										x							x	
waser2014			x			x								x	x			x			
weissenbock2016						x		x												x	
woodbury2017								x									x				
wu2011										x										x	
yanez2017			x					x													x
yumer2015			x										x				x				
zaman2015								x									x				
