# Supplementary material for: Visual Parameter Space Exploration in Time and Space
Source: Comput Graph Forum. 2023 Apr 3;42(6):e14785. doi: 10.1111/cgf.14785 (PMC10947302; doi:10.1111/cgf.14785)
Supplement: Supplementary file 1 — Supporting Information [file CGF-42-0-s001.zip › RTA_Papers.pdf]

Papers included in Thematic Analysis

citekey	*title*	*year*	*outlet*	*identifier*
bruckner2010	Result-Driven Exploration of Simulation Parameter Spaces for Visual Effects Design	2010	IEEE TVCG	10.1109/TVCG.2010.190
waser2010	World Lines	2010	IEEE TVCG	10.1109/TVCG.2010.223
malik2010	Comparative Visualization for Parameter Studies of Dataset Series	2010	IEEE TVCG	10.1109/TVCG.2010.20
kerr2010	Toward evaluating material design interface paradigms for novice users	2010	ACM TOG	10.1145/1778765.1778772
matkovic2010a	Interactive Visual Analysis of Multiple Simulation Runs Using the Simulation Model View: Understanding and Tuning of an Electronic Unit Injector	2010	IEEE TVCG	10.1109/TVCG.2010.171
afzal2011	Visual analytics decision support environment for epidemic modeling and response evaluation	2011	VAST	10.1109/VAST.2011.6102457
matejka2018	Dream Lens: Exploration and Visualization of Large-Scale Generative Design Datasets	2018	CHI	10.1145/3173574.3173943
he2020	InSituNet: Deep Image Synthesis for Parameter Space Exploration of Ensemble Simulations	2020	IEEE TVCG	10.1109/TVCG.2019.2934312
bernard2019	Visual-Interactive Preprocessing of Multivariate Time Series Data	2019	EG CGF	10.1111/cgf.13698
torsney-weir2011	Tuner: Principled Parameter Finding for Image Segmentation Algorithms Using Visual Response Surface Exploration	2011	IEEE TVCG	10.1109/TVCG.2011.248
umetani2011	Sensitive Couture for Interactive Garment Modeling and Editing	2011	ACM TOG	10.1145/2010324.1964985
pretorius2011	Visualization of Parameter Space for Image Analysis	2011	IEEE TVCG	10.1109/TVCG.2011.253
pretorius2015	Visual parameter optimisation for biomedical image processing	2015	BMC Bioinfor	10.1186/1471-2105-16-S11-S9
bao2013	Generating and exploring good building layouts	2013	ACM TOG	10.1145/2461912.2461977
millward2013	An operational software tool for the analysis of coronagraph images: Determining CME parameters for input into the WSA-Enlil heliospheric model	2013	Wiley Space	10.1002/swe.20024
coffey2013	Design by Dragging: An Interface for Creative Forward and Inverse Design with Simulation Ensembles	2013	IEEE TVCG	10.1109/TVCG.2013.147
chaudhuri2013	Attribit: content creation with semantic attributes	2013	UIST	10.1145/2501988.2502008
bogl2013	Visual Analytics for Model Selection in Time Series Analysis	2013	IEEE TVCG	10.1109/TVCG.2013.222
ribicic2013	Visual Analysis and Steering of Flooding Simulations	2013	IEEE TVCG	10.1109/TVCG.2012.175
holbein2018	Parameter Space Comparison of Inertial Particle Models	2018	VMV	10.2312/vmv.20181254

khan2019	GenYacht: An interactive generative design system for computer-aided yacht hull design	2019	Elsevier Oce	10.1016/j.oceaneng.2019.106462
desai2019	Geppetto: Enabling Semantic Design of Expressive Robot Behaviors	2019	CHI	10.1145/3290605.3300599
orban2019	Drag and Track: A Direct Manipulation Interface for Contextualizing Data Instances within a Continuous Parameter Space	2019	IEEE TVCG	10.1109/TVCG.2018.2865051
swearngin2020	Scout: Rapid Exploration of Interface Layout Alternatives through High-Level Design Constraints	2020	CHI	10.1145/3313831.3376593
umetani2012	Guided Exploration of Physically Valid Shapes for Furniture Design	2012	ACM TOG	10.1145/2185520.2185582
koyama2014	Crowd-powered parameter analysis for visual design exploration	2014	UIST	10.1145/2642918.2647386
luboschik2014	Supporting the integrated visual analysis of input parameters and simulation trajectories	2014	Elsevier Com	10.1016/j.cag.2013.09.004
beham2014	Cupid: Cluster-Based Exploration of Geometry Generators with Parallel Coordinates and Radial Trees	2014	IEEE TVCG	10.1109/TVCG.2014.2346626
sorger2016	LiteVis: Integrated Visualization for Simulation-Based Decision Support in Lighting Design	2016	IEEE TVCG	10.1109/TVCG.2015.2468011
poco2014	Visual Reconciliation of Alternative Similarity Spaces in Climate Modeling	2014	IEEE TVCG	10.1109/TVCG.2014.2346755
doraiswamy2015	Topology-based catalogue exploration framework for identifying view-enhanced tower designs	2015	ACM TOG	10.1145/2816795.2818134
ruppert2014	Visual access to an agent-based simulation model to support political decision making	2014	i-KNOW	10.1145/2637748.2638410
matkovic2014	Visual Analytics for Complex Engineering Systems: Hybrid Visual Steering of Simulation Ensembles	2014	IEEE TVCG	10.1109/TVCG.2014.2346744
weissenbock201	PorosityAnalyzer: Visual analysis and evaluation of segmentation pipelines to determine the porosity in fiber-reinforced polymers	2016	VAST	10.1109/VAST.2016.7883516
yumer2015	Semantic shape editing using deformation handles	2015	ACM TOG	10.1145/2766908
frohler2016	GEMSe: Visualization-Guided Exploration of Multi-channel Segmentation Algorithms	2016	EG CGF	10.1111/cgf.12895
obermaier2016	Visual Trends Analysis in Time-Varying Ensembles	2016	IEEE TVCG	10.1109/TVCG.2015.2507592
luboschik2015	Feature-Driven Visual Analytics of Chaotic Parameter-Dependent Movement	2015	EG CGF	10.1111/cgf.12654

berseth2021	Interactive Architectural Design with Diverse Solution Exploration	2021	IEEE TVCG	10.1109/TVCG.2019.2938961
walch2020	LightGuider: Guiding Interactive Lighting Design using Suggestions, Provenance, and Quality Visualization	2020	IEEE TVCG	10.1109/TVCG.2019.2934658
liu2018b	Understanding the Relationship Between Interactive Optimisation and Visual Analytics in the Context of Prostate Brachytherapy	2018	IEEE TVCG	10.1109/TVCG.2017.2744418
hazarika2020	NNVA: Neural Network Assisted Visual Analysis of Yeast Cell Polarization Simulation	2020	IEEE TVCG	10.1109/TVCG.2019.2934591
liu2021b	Supporting the Problem-Solving Loop: Designing Highly Interactive Optimisation Systems	2021	IEEE TVCG	10.1109/TVCG.2020.3030364
bernard2018	Combining the automated segmentation and visual analysis of multivariate time series	2018	EuroVA	10.2312/eurova.20181112
schulz2017	Interactive design space exploration and optimization for CAD models	2017	ACM TOG	10.1145/3072959.3073688
umetani2014	Pteromys: interactive design and optimization of free-formed free-flight model airplanes	2014	ACM TOG	10.1145/2601097.2601129
cibulski2017	Super-Ensembler: interactive visual analysis of data surface sets	2017	SCCG	10.1145/3154353.3154362
eichner2020	Making Parameter Dependencies of Time-Series Segmentation Visually Understandable	2020	EG CGF	10.1111/cgf.13894
biswas2017	Visualization of Time-Varying Weather Ensembles across Multiple Resolutions	2017	IEEE TVCG	10.1109/TVCG.2016.2598869
steiner2017	Integrated Structural–Architectural Design for Interactive Planning	2017	EG CGF	10.1111/cgf.12996
gunther2016a	Inertial Steady 2D Vector Field Topology	2016	EG CGF	10.1111/cgf.12846
matkovic2017	Quantitative Externalization of Visual Data Analysis Results Using Local Regression Models	2017	CD-MAKE	10.1007/978-3-319-66808-6_14
schwarzl2019	Cellpackexplorer: Interactive model building for volumetric data of complex cells	2019	Elsevier Com	10.1016/j.cagx.2019.100010
zaman2015	GEM-NI: A System for Creating and Managing Alternatives In Generative Design	2015	CHI	10.1145/2702123.2702398
ribes2019	A Visual Sensitivity Analysis for Parameter-Augmented Ensembles of Curves	2019	ASME Journa	10.1115/1.4046020
unger2012	A Visual Analysis Concept for the Validation of Geoscientific Simulation Models	2012	IEEE TVCG	10.1109/TVCG.2012.190
konev2014	Run Watchers: Automatic Simulation-Based Decision Support in Flood Management	2014	IEEE TVCG	10.1109/TVCG.2014.2346930
